# Systemic Lupus Erythematous and Malignancy Risk: A Meta-Analysis

**DOI:** 10.1371/journal.pone.0122964

**Published:** 2015-04-17

**Authors:** Lihong Cao, Hongyan Tong, Gaixiang Xu, Ping Liu, Haitao Meng, Jinghan Wang, Xiaoying Zhao, Yongmin Tang, Jie Jin

**Affiliations:** 1 Department of Hematology, the First Affiliated Hospital of Zhejiang University, Hangzhou, People’s Republic of China; 2 Institute of Hematology, Zhejiang University School of Medicine, Hangzhou, People’s Republic of China; 3 School of Population Health, the University of Western Australia, Perth, Australia; 4 Department of Hematology, the Second Affiliated Hospital of Zhejiang University, Hangzhou, People’s Republic of China; 5 Department of Hematology, the Affiliated Children’s Hospital of Zhejiang University, Hangzhou, People’s Republic of China; Baylor College of Medicine, UNITED STATES

## Abstract

**Background:**

Pilot studies have estimated cancer incidence in patients with systemic lupus erythematous (SLE). However, the results have been inconclusive. To ascertain the correlation between SLE and malignancy more comprehensively and precisely, we conducted a meta-analysis.

**Methods:**

PubMed, the Cochrane Library and Embase databases through June 2014, were searched to identify observational studies evaluating the association between SLE and malignancy. The outcomes from these studies were measured as relative risks (RRs). A random or fixed effects model was chosen to calculate the pooled RR according to heterogeneity test. Between-study heterogeneity was assessed by estimating I^2^ index. Publication bias was assessed by Egger’s test.

**Results:**

A total of 16 papers, including 59,662 SLE patients, were suitable for the meta-analysis. Of these papers, 15 reported RRs for overall malignancy, 12 for non-Hodgkin lymphoma (NHL) and lung cancer, 7 for bladder cancer, 6 for Hodgkin lymphoma (HL) and leukemia, 5 for skin melanoma, and liver and thyroid cancers, 4 for multiple myeloma (MM), and esophageal and vaginal/vulvar cancers and 3 for laryngeal and non-melanoma skin cancers. The pooled RRs were 1.28 (95% CI, 1.17–1.41) for overall cancer, 5.40 (95% CI, 3.75–7.77) for NHL, 3.26(95% CI, 2.17–4.88) for HL, 2.01(95% CI, 1.61–2.52) for leukemia, 1.45(95% CI, 1.04–2.03) for MM, 4.19(95% CI, 1.98–8.87) for laryngeal cancer, 1.59 (95% CI, 1.44–1.76) for lung cancer, 1.86(95% CI, 1.21–2.88) for esophageal cancer, 3.21(95% CI, 1.70–6.05) for liver cancer, 3.67(95% CI, 2.80–4.81) for vaginal/vulvar cancer, 2.11(95% CI, 1.12–3.99) for bladder cancer, 1.51(95% CI, 1.12–2.03) for non-melanoma skin cancer, 1.78(95% CI, 1.35–2.33) for thyroid cancer, and 0.65(95% CI, 0.50–0.85) for skin melanoma. Only the meta-analyses of overall malignancy, NHL, and liver and bladder cancers produced substantial heterogeneity (I^2^, 57.6% vs 74.3% vs 67.7% vs 82.3%). No apparent publication bias was detected except for NHL studies.

**Conclusions:**

Our data support an association between SLE and malignancy, not only demonstrating an increased risk for NHL, HL, leukemia, and some non-hematologic malignancies, including laryngeal, lung, liver, vaginal/vulvar, and thyroid malignancies, but also a reduced risk for skin melanoma. Although an increased risk of MM, and esophageal, bladder and non-melanoma skin cancers was identified from the accumulated data in these studies, this observation requires confirmation.

## Introduction

Systemic lupus erythematous (SLE) is one of the most common systemic autoimmune rheumatic diseases. It often affects multiple organ systems and has a broad range of clinical and laboratory manifestations[1–3] and occasionally co-exists with Sjogren’s syndrome or other overlapping syndromes [4]. Systemic glucocorticoids are used alone or combined with other immunosuppressive or cytotoxic agents including methotrexate, cyclophosphamide, and azathioprine to treat patients with significant organ involvement or refractory symptoms [5,6]. Although survival in SLE has improved since the introduction of new biological drugs such as rituximab[5], there continues to be significant morbidity like cancer adversely affecting long-term outcome.

Intrinsic immune system defects in SLE, combined with exposure to cytotoxic medications, foster the emergence of site-specific cancers [6–8]. In the past decades, quite a few studies have investigated the link between SLE and malignancy [9–24]. A meta-analysis first confirmed that SLE is a risk factor for NHL in 2005 [6]. However, there have been additional observational studies questioning the strength of the evidence for such an association since that time [19–24]. To ascertain the risks of overall and site-specific malignancies in patients with SLE more comprehensively and precisely, we conducted a meta-analysis.

## Methods

### Search Strategy for Identification of Studies

We searched PubMed, the Cochrane Library and Embase databases through June 2014 for English articles using the following words (all fields), which were retrieved from all eligible articles and the references were reviewed to identify additional relevant studies: [‘autoimmune diseases’ OR ‘systemic lupus erythematous’ OR ‘SLE’] and [‘lymphoma’ OR ‘malignancy’ OR ‘cancer’ OR ‘neoplasm’ OR ‘tumor’] AND [‘case-control’ OR ‘cohort’].

### Inclusion and Exclusion Criteria

Eligible studies fulfilled the following criteria: (1) case-control or cohort study; (2) SLE as one of the exposure interests; (3) cancer as one outcome of interest; (4) general population as the control group; (5) relative risk (RR), standardized incidence rate (SIR), or standardized morbidity rate (SMR) with 95% confidence interval (CI) available (or data available for calculations). Reviews [3,7,25–27] or editorials, letters to the editor without original data, and case reports were excluded. In the event of multiple publications from the same study or overlapping study populations, only the most relevant one was selected.

### Data Extraction

Data extraction was conducted by one investigator (L-HC) and checked by two investigators (G-XX and PL). Discrepancies were resolved through team consensus. The collected data included general information (design type, author, published year, country, study period, and follow-up), cohort of SLE characteristics (gender, mean age at the time of SLE diagnosis, diagnostic criteria, exclusion criteria, use of immunosuppressive drugs, and adjusted variables), and results (number of participants, reference population, and RR, SIR, or SMR with corresponding 95% CI). The quality of each study was evaluated independently by the above three authors using the Newcastle-Ottawa scale[28](S1 Table).

### Data Synthesis

The preferred method of data presentation for cohort studies are the calculated RRs compared with the general population, generally estimating the age- and gender-adjusted SIRs or SMRs. SIRs or SMRs are estimated as the ratio of the observed over the expected number of cases for an exposed population. The expected number is calculated as the product of gender-, age-, and calendar period-specific person-years of follow-up, and the corresponding incidence of malignancy in the general population. The 95% CI is calculated assuming a Poisson distribution for the observed cases.

All analyses were performed with Stata 12.0 software. Fixed (Mantel-Haenszel) or random effects (DerSimonian-Laird) models were used to calculate the pooled effect estimates[29]. Between-study heterogeneity was assessed by estimating Ι^2^ (significance level at P <0.1) [52]. In the case of significant heterogeneity, irrespective of the Ι^2^ estimation, random effects models were used. The Galbraith plot was used to detect potential source of heterogeneity [30]. We also performed subgroup analyses with data available. In addition, sensitivity analyses were carried out by excluding specific studies. Egger’s tests were used to evaluate publication bias for all meta-analyses [31]. The presence of publication bias was ascertained if the P value was <0.05.

## Results

### Characteristics of the Included Studies

**Fig 1:**
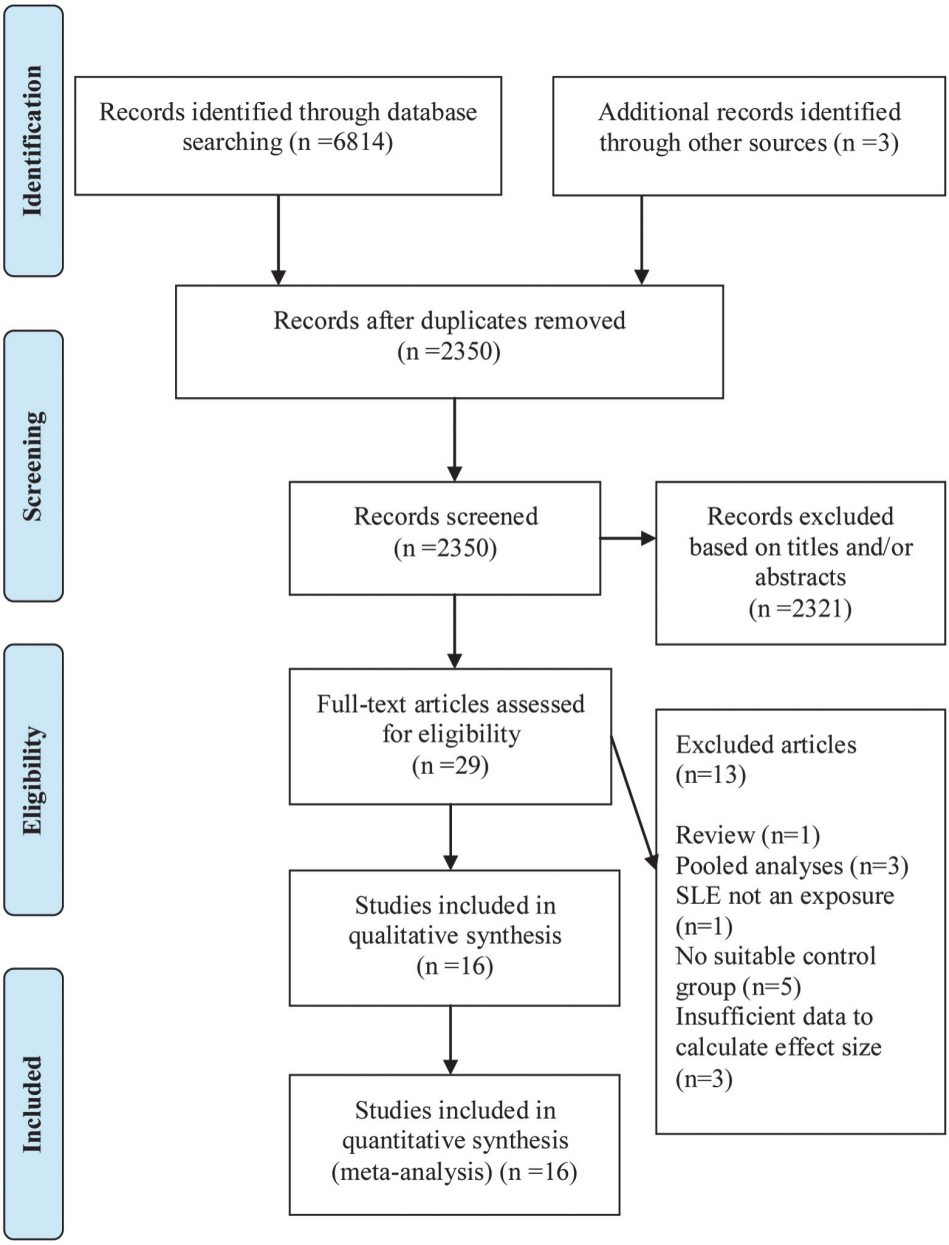
PRISMA Flow Diagram.

A total of 2,350 articles met the defined search criteria, the titles and/or abstracts of which were screened to identify the potentially relevant articles. Of them, 29 publications were further analyzed. Finally, 16 cohort studies were selected for the meta-analysis (Fig 1). All of the studies were published between 1992 and 2013. Eight studies were performed in Europe, five in North America, two in Korea, and one international multi-center cohort study from the USA, Canada, Europe, and Korea. These studies included population- and hospital-based SLE cohorts that ranged from 116–30,478 patients and had a mean follow-up time of 4.8–13.4 years (Table 1).

**Table 1:** Characteristics of selected SLE cohort studies.

Source (published year, country)	Study period	Study Type	Cohort of SLE					Follow-up: Mean /patient-years	Adjustment for covariates
			N/ Female, %	Mean age at diagnosis of SLE (y)	Diagnostic criteria	Exclusion Criteria	Immunosuppressive Drugs		
Pettersson[9] (1992, Finland)	1967–1987	Hospital-based	205 /88.8	NR	ARA (1971)	Patients with discoid or drug induced lupus	2 cases with CTX	NR /2340	Age and sex
Sweeney[10] (1995, USA)	1981–1991	Population-based	219 /100	NR	ACR (1982)	Not excluding overlap syndrome	1 NHL before entry into this study treated with FK-506 following renal transplant	5.2 /NR	Age, sex and race
Abu-Shakra[11] (1996, Canada)	NR	Population-based	724 /86.6	33.3 (8–83.1)	ACR (1982)	All antecedent cancers	None NHLs	24 (duration) /7,233	Age and sex
Mellemkjaer [12] (1997, Denmark)	1977–1989	Hospital-based	1,585 /83	NR	NR	Cancers diagnosed in the first year of SLE	7 Cases with AZA	6.8/10,807	Age, sex, and calendar time at diagnosis
Ramsey-Goldman[13] (1998, USA)	1985–1995	Population-based	616/100	NR	ACR (1982)	NR	NR	≥15(duration) /NR	Age, gender and race
Sultan[14] (2000, UK)	1978–1999	Hospital-based	276 /93.5	34.7	ACR (1982)	Patients with discoid lupus and drug induced lupus	18% SLEs and 1 case with CTX	4.8 /1,695	Age and sex
Cibere[15] (2001, Canada)	1975–1994	Population-based	297 /84	36 (10–80)	ACR (1982)	Cancers diagnosed after the diagnosis of SLE but before follow-up	25% SLEs with AZA, 8% with CTX, 6% with MTX	12 /3,587	Age, sex, and calendar time at diagnosis
Nived[16] (2001, Swenden)	1981–1996	Population-based	116 /NR	48	ACR (1982)	NR	19% SLEs with AZA and/or CTX	9.4 /1,086	Age and sex
Bjornadal[17] (2002, Swenden)	1964–1995	Hospital-based	5,715 /74	≥20	NR	Overlap syndrome; Cancers diagnosed in the first year of SLE	NR	8.8 /50,246	Age and sex
Ragnarsson[18] (2003, Iceland)	1957–2001	Population-based	238 /89.5	43.2 (10–81)	ACR (1982)	Patients with discoid lupus and drug induced lupus	NR	NR /2774	Age, sex, and calendar time at diagnosis
Chun[19] (2005, Korea)	1992–2002	Population-based	434 /100	NR	ACR (1997)	NR	NR	NR/ 1673.4	Age, sex, and calendar time at diagnosis
Tarr[20] (2007, Hungary)	1970–2004	Population-based	860 /89.7	33 (16–64)	ACR (1982) /ARA (1971)	NR	76% SLEs with immunosuppressive drugs	13.4 /14,190	Age, sex, and calendar time at diagnosis
Parikh-Patel[21] (2008, USA)	1991–2002	Hospital-based	30,478 /89.0	NR	NR	NR	NR	5.18 /157,969	Age, sex, and calendar time at diagnosis
Kang[22] (2010, Korea)	1997–2007	Population-based	914 /100	29.1	ACR (1982)	Overlap syndrome	37.6% SLEs with AZA, 20.7% with CTX	6.3 /5,716	Age, sex, and race
Dreyer[23] (2011, Denmark)	1943–2006	Hospital-based	576 /88	33 (9–81)	ACR (1982)	Cancers diagnosed in the first year of SLE	NR	13.2 /7,803	Age, sex, and calendar time at diagnosis
Bernatsky[24] (2013, Multisite)	1958–2009	Population-based	16,409 /90	NR	ACR (1982)	Cancers diagnosed in the first year of SLE	NR	7.4 /121,283	Age, sex, and calendar time at diagnosis

**NOTE:** ACR, revised American College of Rheumatology criteria; ARA, 1971 criteria of American Rheumatism Association; AZA, Azathioprine; CTX, Cyclophosphamide; MTX, Methotrexate; N, number of patients with SLE; NR, not reported

### SLE and Risk of Overall Malignancy

**Fig 2:**
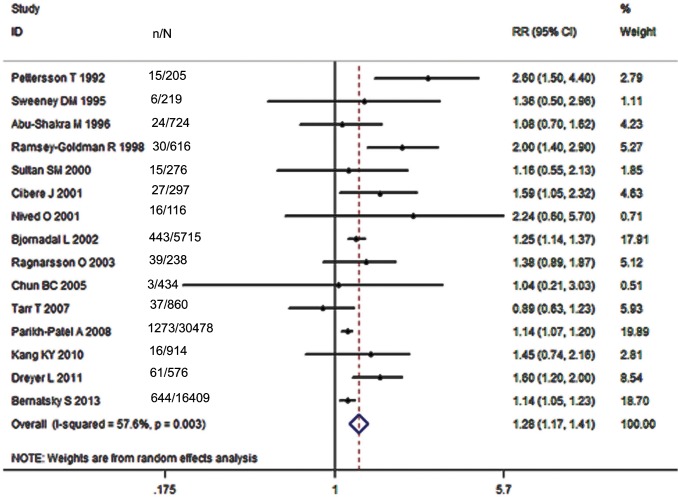
Relative risk of overall malignancy in patients with SLE compared with the general population. (N, number of patients with SLE; n, number of cancer cases).

**Fig 3:**
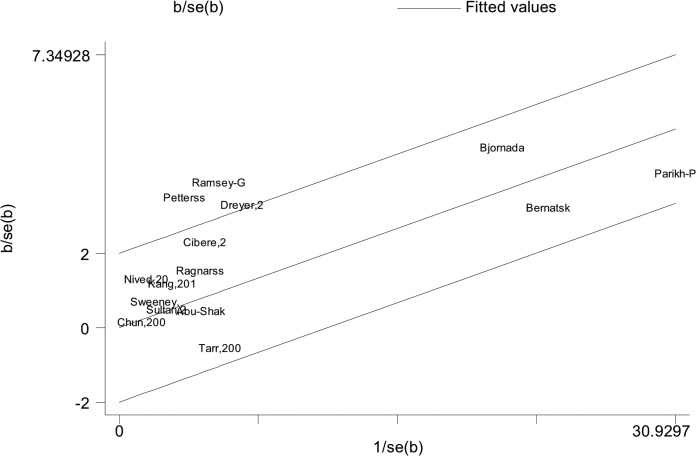
Galbraith plots of association between SLE and overall malignancy.

**Fig 4:**
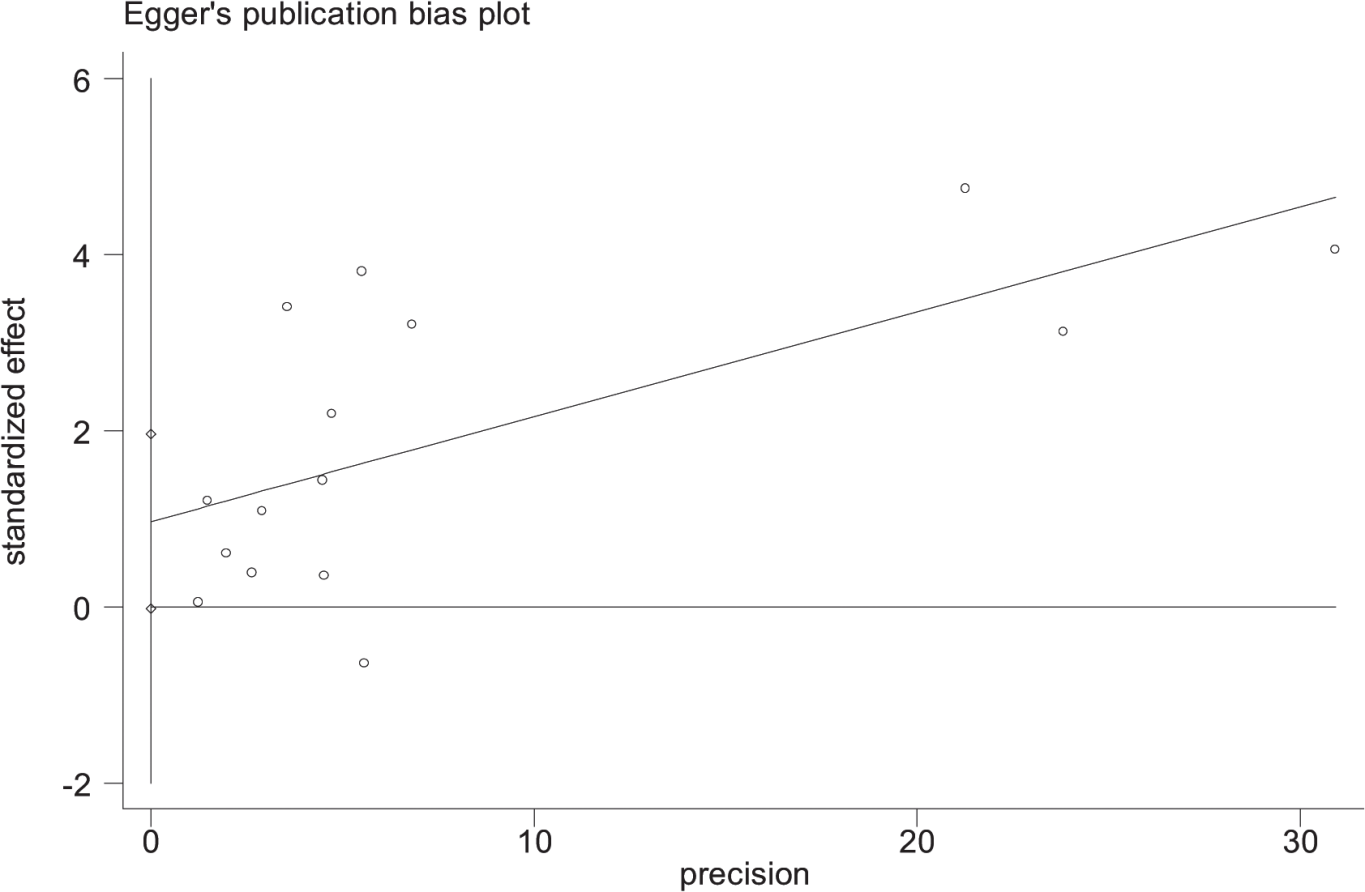
Egger’s test for the publication bias among overall malignancy studies. (P = 0.055).

Of all included studies, fifteen, involving 58,077 patients with SLE, estimated RRs for overall malignancy. As shown in Fig 2, RR of each study ranged from 0.89–2.60 and the pooled RR by random effects analysis was 1.28 (95% CI, 1.17–1.41), with substantial heterogeneity (Ι^2^ = 57.6%). The Galbraith plot showed that three studies might be the major source of the heterogeneity (Fig 3), two conducted in Europe [9,23] and one in North America [13]. The regional subgroup analysis suggested that the European studies, as well as North American ones produced substantial heterogeneity (Ι^2^, 61.3% vs. 65.6%; Table 2). The pooled RRs in two areas were equally 1.37 (Table 2 and S1 Fig). We also performed subgroup analyses by study type, diagnostic criteria for SLE, whether cancers diagnosed in the first year of SLE being excluded, gender, and SLE duration (Table 2). As shown in S2 Fig, a higher risk of overall cancer was noted in the hospital-based cohorts (pooled RR, 1.33; 95% CI, 1.14–1.55) compared with the population-based ones (pooled RR, 1.29; 95% CI, 1.09–1.53). Considerable heterogeneity was found in the hospital-based subgroup (Ι^2^ = 75.8%), moderate heterogeneity in the population-based one (Ι^2^ = 45.4%). The studies adopting the American College of Rheumatology criteria for the classification of SLE (ACR, 1982)[32] conferred a lower risk to develop a cancer (pooled RR, 1.40; 95% CI, 1.19–1.65) than the study with 1971 criteria of American Rheumatism Association (ARA, 1971)[33] did (RR, 2.6; 95% CI, 1.5–4.4; S3 Fig). The cohorts with ACR criteria produced substantial heterogeneity (Ι^2^ = 50.8%). When cancers diagnosed in the first year of SLE were excluded, the summary risk estimate was 1.25(95% CI, 1.10–1.43), otherwise, it increased (1.36; 95% CI, 1.14–1.63; S4 Fig). Both subgroups produced substantial heterogeneity (Ι^2^, 72.3% vs 56.6%). Males were at a higher risk to develop a cancer (pooled RR, 2.41; 95% CI, 1.46–3.98) than females (pooled RR, 1.62; 95% CI, 1.36–1.94; S5 Fig). Neither gender subgroup produced heterogeneity. Only the study [23] demonstrated that SLE population ≥ 50 years of age had a 1.60-fold cancer risk, the same as those <50 years of age. Two studies [23,24] evaluated SLE-duration-specific RRs and the pooled RRs were as follows: 2.21(95% CI, 1.71–2.85) for <1 y; 1.26 (95% CI, 1.07–1.47) for 1–4 y; 1.16 (95% CI, 0.86–1.55) for 5–9 y; 1.12 (95% CI, 0.89–1.41) for 10–19 y; and 1.43 (95% CI, 0.65–3.11) for 20+ y. There wasn’t heterogeneity between the two studies when it was less than 5 years since the diagnosis of SLE. The heterogeneity was moderate when it was 5–19 years. Considerable heterogeneity emerged when it was more than 20 years (Ι^2^ = 87.7%). Egger’s test was used to evaluate publication bias for the meta-analysis of overall malignancy. There was no apparent asymmetric distribution occurring in it (P = 0.055; Fig 4). When sensitivity analysis for the meta-analysis was executed, no significant change in pooled RR was found by sequential omission of individual studies, indicating that the result was stable and reliable.

**Table 2:** Stratified analyses of pooled relative risks of overall malignancy in patients with SLE.

Subgroups	Number of studies	References	N	Pooled RR or RR(95% CI)	Heterogeneity	
					I^2^ (%)	p Value
Study type						
Hospital-based	5	[9,14,17,21,23]	37,250	1.33(1.14–1.55)	75.8	0.002
Population-based	10	[10–11,13,15–16,18,20,22,24]	20,827	1.29(1.09–1.53)	45.4	0.058
Region						
Europe	7	[9,14,16–18,20,23]	7,986	1.37(1.11–1.69)	61.3	0.017
North America	5	[10–11,13,15,21]	32,334	1.37(1.06–1.77)	65.6	0.020
Asia	2	[19,22]	1,348	1.38(0.84–2.28)	0.0	0.651
Multisite	1	[24]	16,409	1.14(1.05–1.23)	-	-
SLE diagnostic criteria						
ARA,1971	1	[9]	205	2.6(1.5–4.4)	-	-
ACR,1982	10	[10–11,13–16,18,22–24]	20,385	1.40(1.19–1.65)	50.8	0.032
Excluding cancers diagnosed in the first year of SLE						
Yes	3	[17,23,24]	22,700	1.25(1.10–1.43)	72.3	0.027
No	12	[9–11,13–16,18–22]	35,377	1.36(1.14–1.63)	56.6	0.008
Gender						
Male	4	[9,11,16,23]	>189,<305	2.41(1.46–3.98)	0.0	0.909
Female	8	[9–11,13,16,18–19,23]	>2,585,<2,701	1.62(1.36–1.94)	0.0	0.582
Age						
≥50 y	1	[23]	576	1.60(1.21–2.12)	-	-
<50 y	1	[23]	576	1.60(0.99–2.60)	-	-
Time since SLE diagnosis						
<1 y	2	[23,24]	16,985	2.21(1.71–2.85)	0.0	0.930
1–4 y	2	[23,24]	16,985	1.26(1.07–1.47)	0.0	0.893
5–9 y	2	[23,24]	16,985	1.16(0.86–1.55)	37.8	0.205
10–19 y	2	[23,24]	16,985	1.12(0.89–1.41)	30.8	0.229
>20 y	2	[23,24]	16,985	1.43(0.65–3.11)	87.7	0.004

**NOTE:** ACR, revised American College of Rheumatology criteria; ARA, 1971 criteria of American Rheumatism Association; N, number of patients with SLE

### SLE and NHL Risk

**Fig 5:**
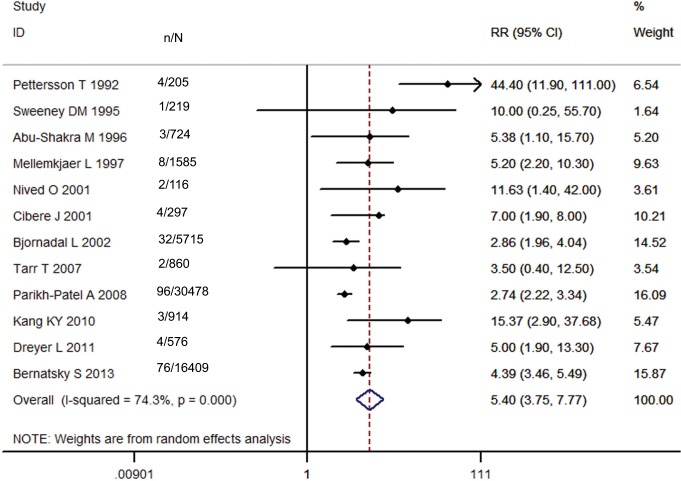
Relative risk of NHL in patients with SLE compared with the general population. (N, number of patients with SLE; n, number of cancer cases.)

**Fig 6:**
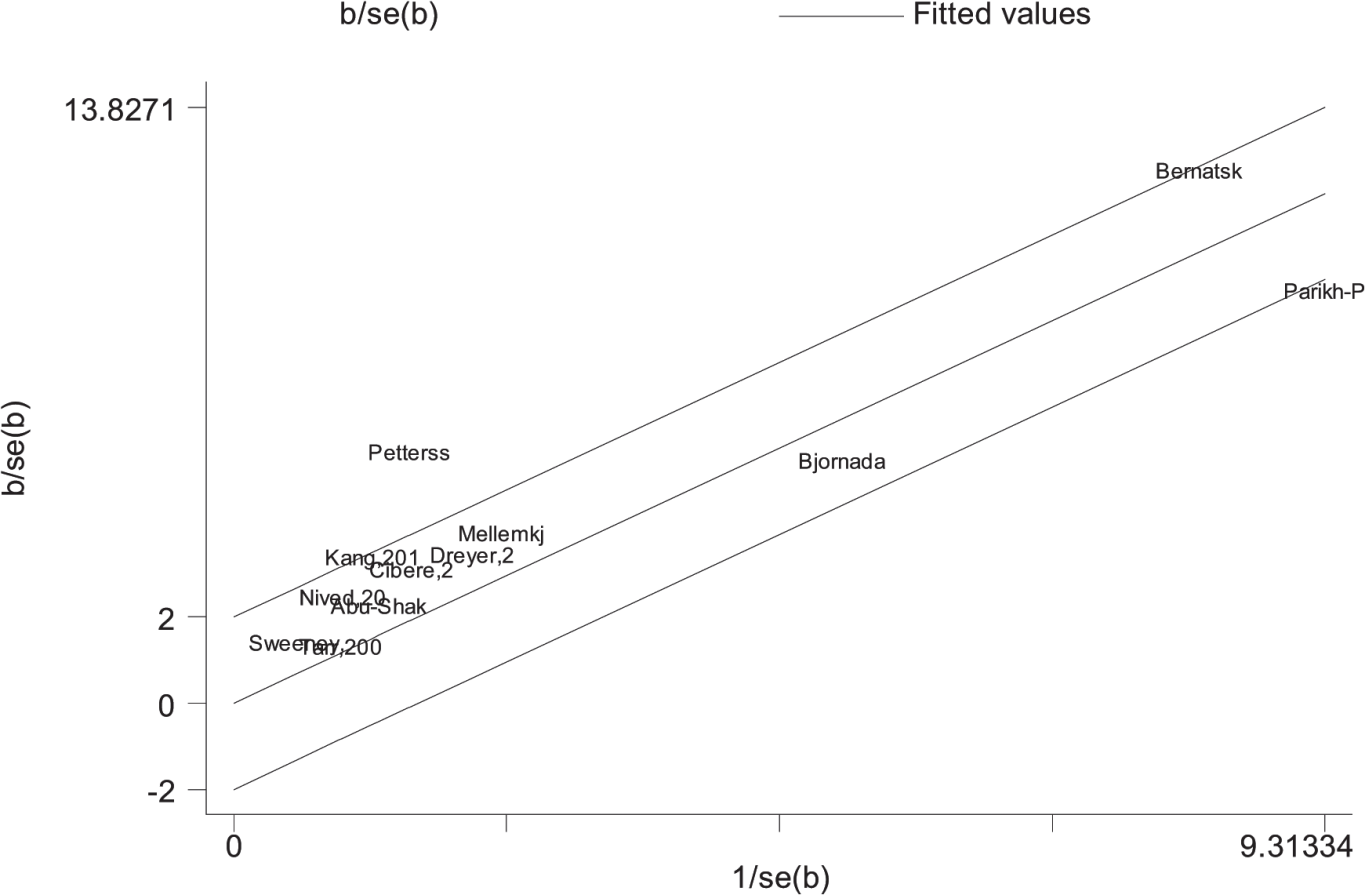
Galbraith plots of association between SLE and NHL.

**Fig 7:**
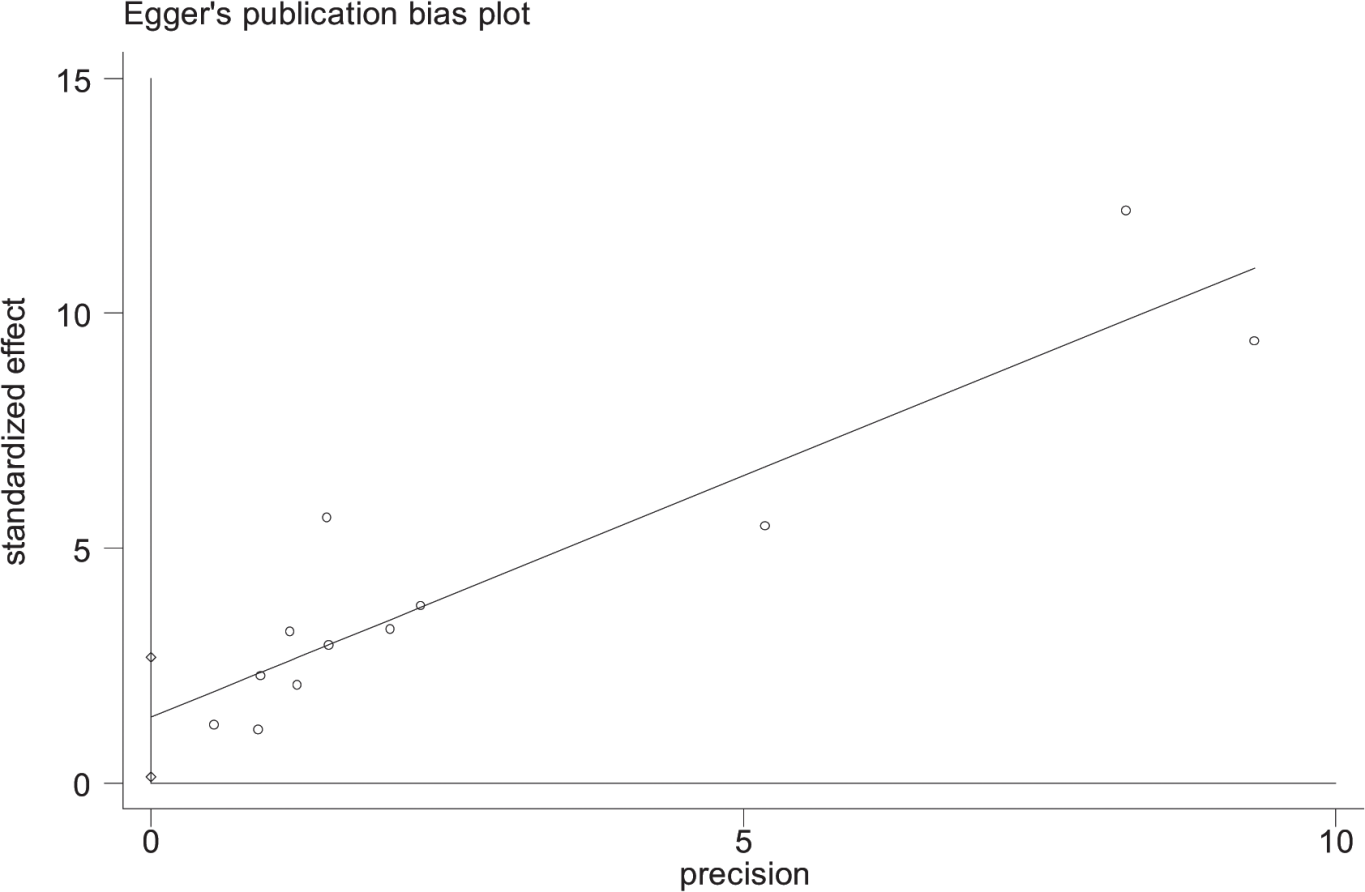
Egger’s test for the publication bias among NHL studies. (P = 0.035).

As shown in Fig 5, twelve studies, involving 58,098 SLE patients, demonstrated an increased incidence of NHL with the exception of one study [20]. Of them, one study [9] revealed a dramatically elevated RR (44.40; 95% CI, 11.90–111.00). The pooled RR of all studies was 5.40 (95% CI, 3.75–7.77), but with substantial heterogeneity (Ι^2^ = 74.3%). The possible source of heterogeneity might be attributed to two studies [9,21], one performed in Finland and the other in the USA, according to the Galbraith plot (Fig 6). The regional subgroup analysis confirmed the European studies produced considerable heterogeneity (Ι^2^ = 78.5%) while the North American studies didn’t (Table 3). The pooled RRs for two areas were 6.74 (95% CI, 2.98–15.25) and 7.86 (95% CI, 4.52–13.70), respectively (S1 Fig). One Asian study reported a RR of 15.37 with wide 95% CI (2.90–37.68). One international multicenter study [24] with 16,409 lupus patients presented a RR of 4.39 (95% CI, 3.46–5.49). The results of the other subgroup analyses were shown in Table 3. A higher risk was observed in the hospital-based subgroup than in the population-based one (pooled RR, 7.94 vs 5.06; S2 Fig). Considerable heterogeneity was present in the hospital-based cohorts (Ι^2^ = 84.5%). The heterogeneity among the population-based studies (Ι^2^ = 4.4%) might not be important. In the analysis stratified by diagnostic criteria for SLE, the risk of NHL (pooled RR and 95% CI) was significantly decreased in the subgroup with ACR criteria, 4.97 (3.94–6.27), compared to that with ARA criteria (S3 Fig). The heterogeneity in the subgroup with ACR criteria might not be important (Ι^2^ = 2.3%). When NHLs in the first year of SLE were excluded, the summary risk estimate was 3.92(95% CI, 2.97–5.18) with moderate heterogeneity (Ι^2^ = 34.4%), otherwise, it increased (8.15, 95% CI, 3.63–18.32) with considerable heterogeneity (Ι^2^ = 81.0%; S4 Fig). Only one study [12] demonstrated that females had a lower risk to develop NHL than males (RR, 4.1 vs. 9.4). The same study estimated RRs by different age groups (12.8 for 0–39 y; 8.1 for 40–59 y; 3.7 for >60 y) and length of follow-up (2.21 for <1 y; 1.26 for 1–4 y; 1.16 for 5–9 y; 1.12 for 10–19 y; 1.43 for >20 y). Egger’s test showed that Publication bias was potentially present in the meta-analysis of NHL (P = 0.035; Fig 7). Sensitivity analysis confirmed that the risk estimate for NHL was stable and reliable.

**Table 3:** Stratified analyses of pooled relative risks of NHL in patients with SLE.

Subgroups	Numberof studies	References	N	Pooled RR orRR(95% CI)	Heterogeneity	
					I^2^ (%)	p Value
Study type						
Hospital-based	5	[9,12,17,21,23]	38,559	7.94(3.16–19.95)	84.5	0.000
Population-based	7	[10–11,15–16,20,22,24]	19,539	5.06(3.89–6.58)	4.4	0.393
Region						
Europe	6	[9,12,16–17,20,23]	9,057	6.74(2.98–15.25)	78.5	0.000
North America	4	[10,11,15,21]	31,718	7.86(4.52–13.70)	0.0	0.684
Asia	1	[22]	914	15.37(2.90–37.68)	-	**-**
Multisite	1	[24]	16,409	4.39(3.46–5.49)	-	-
SLE diagnostic criteria						
ARA,1971	1	[9]	205	44.4(11.9–111)	-	-
ACR,1982	7	[10–11,15–16,22–24]	19,255	4.97 (3.94–6.27)	2.3	0.407
Excluding cancers diagnosed in the first year of SLE						
Yes	4	[12,17,23,24]	24,285	3.92(2.97–5.18)	34.4	0.206
No	8	[9–11,15–16,20–22]	33,513	8.15(3.63–18.32)	81.0	0.000
Gender						
Female	1	[12]	1,316	4.1(1.3–9.6)	-	-
Male	1	[12]	269	9.4(1.9–27)	-	-
Age						
0–39 y	1	[12]	1,585	12.8(0.3–17.5)	-	-
40–59 y	1	[12]	1,585	8.1(1.7–24)	-	-
>60 y	1	[12]	1,585	3.7(1.0–9.4)	-	-
Length of follow-up						
<1 y	1	[12]	1,585	16.8(3.5–49)	-	-
1–4 y	1	[12]	1,585	1.6(0–8.7)	-	-
5–9 y	1	[12]	1,585	3.9(0.5–14)	-	-
10–15 y	1	[12]	1,585	10.1(1.2–36)	-	-

**NOTE:** ACR, revised American College of Rheumatology criteria; ARA, 1971 criteria of American Rheumatism Association; N, number of patients with SLE

### SLE and the Risk for Other Site-specific Malignancies

As shown in Table 4, there was a direct association between SLE and three hematologic malignancies. The meta-analyses of HL and leukemia both included 54,760 lupus patients from six studies, with the summary estimates of 3.26(95% CI, 2.17–4.88) and 2.01(95% CI, 1.61–2.52), respectively (S6 and S7 Figs). The meta-analysis of MM involved 54,187 patients with SLE from four studies, demonstrating a pooled RR of 1.45(95% CI, 1.04–2.03; S8 Fig). None of the three meta-analyses had heterogeneity and publication bias. The sensitivity analyses for HL and leukemia confirmed that their risk estimates were stable and reliable. However, if the studies [21, 24] were omitted sequentially, the pooled RRs for MM would be 1.30(95%, 0.87–1.94) and 1.54(95% CI, 0.98–2.42), which showed that the risk estimate for MM was potentially unstable and unreliable.

**Table 4:** Pooled relative risks of site-specific malignancies in patients with SLE.

Cancer site	Numberof studies	References	n/N	Pooled RR(95% CI)	Heterogeneity		Egger’s test(P value)
					I^2^ (%)	p Value	
Hematologic							
HL	6	[12,14–15,17,21,24]	29/54,760	3.26(2.17–4.88)	0.0	0.631	0.145
Leukemia	6	[11–12,17,21,23–24]	85/54,760	2.01(1.61–2.52)	0.0	0.979	0.905
Multiple myeloma	4	[12,17,21,24]	33/54,187	1.45(1.04–2.03)	0.0	0.787	0.927
Respiratory							
Larynx	3	[12,17,18]	9/7,538	4.19(1.98–8.87)	1.3	0.363	0.337
Lung	12	[11–18,20–21,23–24]	388/57,890	1.59(1.44–1.76)	19.4	0.253	0.658
Digestive							
Oropharynx	5	[12,15,17,20,23]	15/9,033	1.54(0.95–2.51)	40.8	0.149	0.902
Esophagus	4	[12,17,21,23]	19/38,354	1.86(1.21–2.88)	0.0	0.831	0.364
Stomach	5	[12,17,20–21,24]	55/55,047	1.16(0.87–1.54)	0.0	0.992	0.215
Colon	4	[11,17–18,23]	39/7,253	1.07(0.77–1.49)	0.0	0.468	0.365
Rectum	2	[17,23]	21/6,291	1.06(0.68–1.67)	0.0	0.875	-
Anus	2	[14,23]	4/852	5.26(0.15–186.61)	84.1	0.012	-
Pancreas	7	[11,12,15,17,21,23–24]	55/55,784	1.21(0.93–1.56)	38.9	0.132	0.395
Liver	5	[12, 17,21,23–24]	53/54,463	3.21(1.70–6.05)	67.7	0.015	0.267
Gynecological							
Ovary	7	[15,17–18,20–21,23–24]	53/54,573	0.76(0.56–1.01)	0.0	0.654	0.631
Vagina/vulva	4	[12,18,21,23]	55/32,877	3.67(2.80–4.81)	39.6	0.174	0.091
Cervix	8	[12,14–15,17,20–21,23–24]	81/56,196	1.39(0.81–2.40)	79.8	0.000	0.304
Uterus	5	[12,17–18,21,24]	73/54,425	0.78(0.50–1.21)	58.4	0.048	0.665
Breast	12	[9,11–15,17–18,20–21,23–24]	465/57,979	0.88(0.72–1.06)	49.7	0.025	0.056
Urological							
Kidney	5	[11,12,17,21,23]	53/39,078	1.59(0.91–2.78)	52.1	0.079	0.615
Prostate	9	[12,14–18,21,23–24]	90/55,690	1.02(0.67–1.55)	56.8	0.018	0.105
Bladder	7	[12,17,20–24]	85/56,537	2.11(1.12–3.99)	82.3	0.000	0.194
Skin							
Melanoma	5	[12,17,21,23–24]	56/54,753	0.65(0.50–0.85)	0.0	0.737	0.952
Non-melanoma	3	[12,17,23]	46/7,876	1.51(1.12–2.03)	23.1	0.273	0.534
Other							
Thyroid	5	[17,18,21,23–24]	57/53,416	1.78(1.35–2.33)	0.0	0.639	0.576
Brain	5	[12,17–18,21,23]	30/38,592	1.29(0.87–1.90)	0.0	0.490	0.625

**NOTE:** n, number of cancer cases; N, number of patients with SLE

As for non-hematologic malignancies, an increased risk was discovered in lupus patients for laryngeal, lung, esophageal, hepatic, vaginal/vulvar, bladder, non-melanoma skin and thyroid malignancies while a decreased risk was uncovered for melanoma skin cancer (Table 4). Twelve studies estimated RRs for lung cancer, 7 for bladder cancer, 5 for hepatic, melanoma skin and thyroid cancers, 4 for esophageal and vaginal/vulvar cancers and 3 for laryngeal and non-melanoma skin cancers. The sample size of these meta-analyses ranged from 7,538 to 57,890. The pooled RRs were 4.19(95% CI, 1.98–8.87) for laryngeal cancer, 1.59 (95% CI, 1.44–1.76) for lung cancer, 1.86(95% CI, 1.21–2.88) for esophageal cancer, 3.21(95% CI, 1.70–6.05) for liver cancer, 3.67(95% CI, 2.80–4.81) for vaginal/vulvar cancer, 2.11(95% CI, 1.12–3.99) for bladder cancer, 1.51(95% CI, 1.12–2.03) for non-melanoma skin cancer, 1.78(95% CI, 1.35–2.33) for thyroid cancer, and 0.65(0.50–0.85) for melanoma skin cancer (S9–S17 Figs). The meta-analyses of esophageal, melanoma skin and thyroid cancers didn’t produce heterogeneity. The heterogeneity in the meta-analyses of laryngeal, lung and non-melanoma skin cancers might not be important. There was moderate heterogeneity among the vaginal/vulvar cancer studies (Ι^2^ = 39.6%), substantial heterogeneity among the liver cancer ones (Ι^2^ = 67.7%), considerable heterogeneity among the bladder cancer ones (Ι^2^ = 82.3%). The Galbraith plot showed that no study contributed to the heterogeneity among the liver cancer studies and one study [22] might be the major source of the heterogeneity among the bladder cancer ones. No publication bias was found in any non-hematologic malignancy. If the study [21] was excluded, the summary risk estimate of esophageal cancer would be 1.89(95%, 0.93–3.83). If the study [23] was omitted, the pooled RRs for bladder cancer (1.93; 95% CI, 0.97–3.84) and skin non-melanoma (1.35; 95% CI, 0.95–1.91) would lose their statistical significance, which suggested that these risk estimates were potentially unstable and unreliable. However, the risk estimates of the other non-hematologic malignancies were confirmed stable and reliable by sensitivity analyses.

From Table 4, we observed not only a higher pooled RR for malignancies involving the oropharynx, stomach, colon, rectum, anus, pancreas, cervix, uterus, kidney, prostate and brain, but also a lower pooled RR for ovarian and breast cancers. However, they are statistically insignificant, indicating that these malignancies have no clear link with SLE, compared with the general population.

## Discussion

The present study is by far an ever-comprehensive meta-analysis of SLE and cancer risk, providing more precise risk estimates of various malignancies including some rarer cancers such as laryngeal and esophageal cancer in SLE. It demonstrates that NHL, HL, leukemia, and laryngeal, lung, liver, vaginal/vulvar, and thyroid malignancies are more frequently observed in lupus patients than in the general population. In addition, there is a potentially increased risk of MM, and esophageal, bladder and non-melanoma skin cancers. Conversely, there is a reduced risk of melanoma skin cancer. Hence, the overall risk of malignancy in SLE is slightly elevated, compared to the general population.

Nevertheless, these findings may be influenced by some confounding factors. The subgroup analyses for overall cancer and NHL have shown that the risk for hospital-based cohorts is higher than population-based ones. A potential persuasive explanation is that hospitalized lupus patients have a more severe condition so that a greater number develop into a cancer. European and North American lupus populations are at almost the same risk for the development of a cancer as Asian populations but at a much lower risk to develop NHL. The SLE patients with ACR criteria confer a lower risk to develop a cancer, especially NHL, than those with ARA criteria, revealing that preliminary criteria(ARA, 1971) probably overestimates the true risk owing to lack of specificity[33]. From the subgroup analyses stratified by whether cancers diagnosed in the first year of SLE being excluded, it is suggested that the selection of early-occurring malignancies, especially NHL, with SLE-like symptoms potentially overestimate the true risk, too. Males have a higher risk to develop a cancer than females. However, the conclusion is unreliable due to a small sample of men, requiring more studies for confirmation. Regarding trends over SLE duration or follow-up, an increased risk is initially apparent, followed by trends for somewhat lower RRs, and sequentially a rebound. The highest risk estimate occurs during the first year since diagnosis of SLE or the beginning of follow-up probably because some SLE patients with symptoms of cancer are included in the original cohorts. The more elevated risk estimate occurs again during >20 years, which potentially contributes to more cases of SLE-associated malignancy developing later in the disease process [23]. Therefore, it is essential to undertake a longer follow-up period to evaluate the association more precisely. The cancer incidence in the age-specific groups is quite similar while the NHL incidence at the age of forty to fifty-nine is higher than the other age-specific groups. Nonetheless, additional studies are needed to provide stronger evidence due to limited studies. In all, whatever subgroup doesn’t change the risk trends of overall malignancy and NHL at all.

There have been some hypothesized explanations for the differences in the risks of certain malignancies in lupus patients versus the general population. Significant concern is that the baseline immune system defects in this disease, combined with exposures to cytotoxic and immunosuppressive medications, may increase malignancy susceptibility [25].

According to our study, lymphoma, especially NHL, is a common malignancy in lupus patients. Possible mechanism may be composed of several aspects. First, diffuse large B cell lymphoma, originating from activated lymphocytes, has been shown to be the most common NHL subtype arising in SLE, suggesting that chronic inflammation might heighten lymphoma risk in autoimmune diseases like SLE [34]. Second, translocations involving the juxtaposition of an oncogene adjacent to an important gene for immune cell function may favor the emergence of a lymphoma [35]. The probability of a translocation is proportional to the rate of lymphocyte proliferation. The autoimmunity of SLE may up-regulate lymphocyte proliferation [24]. This might explain some of the excess lymphoma risk in SLE. Third, the use of immunosuppressive agents may lead to lymphoma by direct mutagenesis or by disturbing immune surveillance [36]. Fourth, the persistence of EBV infection may be a common etiological pathway for both the SLE and the B-cell malignancy [20,37]. As to other hematologic malignancies, there is a 2.01-fold increase for leukemia risk. Persistent cytopenia, prominent dysplasia on bone marrow smears, and azathioprine treatment may be considered as possible triggers for the development of this disease [38]. The etiology of increased incidence for MM is uncertain, needing further investigation.

With respect to non-hematologic malignancies, lung cancer risk is increased by 59% in lupus patients compared to the general population. Two potential issues might explain the association. Firstly, it is confirmed that there is a case history of more pack-years in lupus patients developing lung cancer than the general population with lung cancer [39]. Moreover, tobacco smoking has been demonstrated not only to be an independent risk factor for lung cancer but to affect disease severity of SLE [40]. This might provides additional motivation for smoking cessation in patients with SLE. Secondly, systemic chronic inflammation derived from SLE could lead to the presence of interstitial lung disease, which could cause lung cancer [41]. Regarding liver cancer risk, SLE patients experiences a 3.21-fold increase compared to the general population. It has been suggested that there is a higher prevalence of hepatitis virus infections in lupus patients possibly due to immunologic dysfunction and immunosuppressant therapy [42,43] and hepatitis B or hepatitis C infection has a potential association with primary liver cancer [44]. The elevated risk for vaginal/vulvar cancer in women with SLE may be ascribed to susceptibility of human papillomavirus (HPV) infection [45]. However, an increased risk for cervical cancer, another HPV-associated cancer hasn’t been suggested in our study. It is probably because vagina/vulva cancer may also share a common inflammatory mechanism with SLE [46]. As for the increased risk for bladder cancer, cyclophosphamide is considered to be a traditional factor [22]. Viral infection is another potential cause [23]. In regard to the higher incidence of non-melanoma skin cancer in SLE, sun sensitivity of the skin [47,48] is an established reason [49]. The following mechanism might explain the excess occurrence of thyroid cancer in SLE. There is a direct association between SLE and thyroid autoimmunity [50] and thyroid autoimmunity itself could increase thyroid cancer risk [51]. The mechanism underlying the higher risk for laryngeal and esophageal cancers and the lower risk for skin melanoma in SLE are not apparent, further studies required.

There are two main limitations which should be considered when interpreting the data from the present study. First, the primary limitation is the heterogeneity especially in the meta-analyses of overall malignancy, NHL, and liver and bladder cancers. Although subgroup analyses have indicated that some confounding factors don’t change the risk trends for overall malignancy and NHL, the other factors like SLE management strategies, especially cytotoxic drugs use, coexistence of overlap syndrome, and lifestyle factors such as smoking have been unavailable from some studies so that these subgroup analyses couldn’t be conducted, which may have had an impact on the estimated accuracy. Second, even though no significant evidence is found by egger’s test in all the meta-analyses with the exception of NHL, we are not able to completely exclude the publication bias because we didn’t unveil unpublished data and only collected the published articles in English.

## Conclusions

Despite the undeniable limitations, owing to a vast sample size, our study support an association between SLE and malignancy, not only demonstrating an increased risk for NHL, HL, leukemia, and some non-hematologic malignancies including laryngeal, lung, liver, vaginal/vulvar, and thyroid malignancies, but also a reduced risk for skin melanoma. Although an increased risk of MM, and esophageal, bladder and non-melanoma skin cancers was identified from the accumulated data in these studies, this observation requires confirmation. The findings highlight the importance of follow-up and cancer screening in lupus patients. Future studies should focus on the underlying mechanisms related to the development of site-specific cancers, especially MM, and laryngeal, esophageal and melanoma skin cancers, in patients with SLE and warrant correlation with our epidemiologic data.

## Supporting Information

S1 PRISMA Checklist S1 PRISMA 2009 Checklist. (DOC)

S1 Fig Relative risks of overall cancer and NHL by region in lupus patients compared with the general population. (DOC)

S2 Fig Relative risks of overall cancer and NHL by study type in lupus patients compared with the general population. (DOC)

S3 Fig Relative risks of overall cancer and NHL by diagnostic criteria for SLE in lupus patients compared with the general population. (DOC)

S4 Fig Relative risks of overall cancer and NHL by whether cancers diagnosed in the first year of SLE being excluded in lupus patients compared with the general population. (DOC)

S5 Fig Relative risk of overall cancer by gender in lupus patients compared with the general population. (DOC)

S6 Fig Relative risk of HL in patients with SLE compared with the general population. (DOC)

S7 Fig Relative risk of leukemia in patients with SLE compared with the general population. (DOC)

S8 Fig Relative risk of MM in patients with SLE compared with the general population. (DOC)

S9 Fig Relative risk of laryngeal cancer in patients with SLE compared with the general population. (DOC)

S10 Fig Relative risk of lung cancer in patients with SLE compared with the general population. (DOC)

S11 Fig Relative risk of esophageal cancer in patients with SLE compared with the general population. (DOC)

S12 Fig Relative risk of liver cancer in patients with SLE compared with the general population. (DOC)

S13 Fig Relative risk of vaginal/vulvar cancer in patients with SLE compared with the general population. (DOC)

S14 Fig Relative risk of bladder cancer in patients with SLE compared with the general population. (DOC)

S15 Fig Relative risk of non-melanoma skin cancer cancer in patients with SLE compared with the general population. (DOC)

S16 Fig Relative risk of thyroid cancer cancer in patients with SLE compared with the general population. (DOC)

S17 Fig Relative risk of melanoma skin cancer cancer in patients with SLE compared with the general population. (DOC)

S1 Table Newcastle-Ottawa Quality Assessment Scale for included studies. (DOCX)
