# Supplementary figures and images for: Systemic Lupus Erythematous and Malignancy Risk: A Meta-Analysis
Source: PLoS One. 2015 Apr 17;10(4):e0122964. doi: 10.1371/journal.pone.0122964 (PMC4401738; doi:10.1371/journal.pone.0122964)

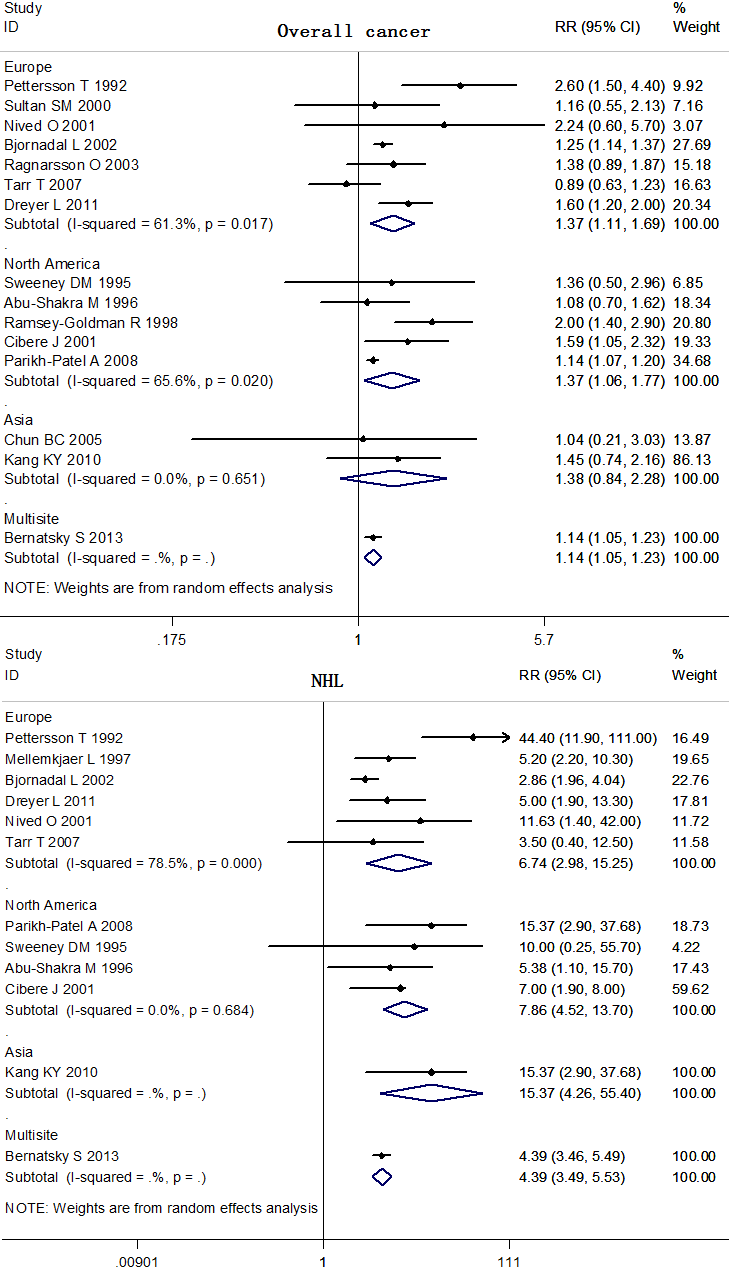

Supplement: S1 Fig — (DOC) [file pone.0122964.s002.doc]

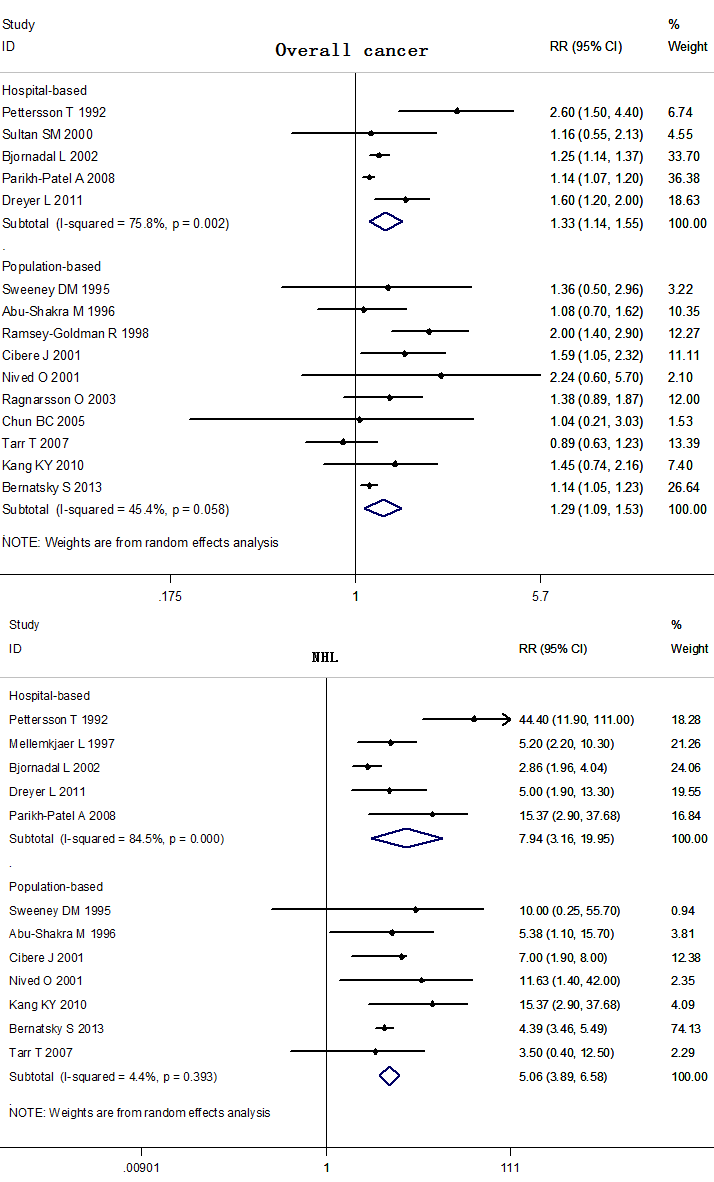

Supplement: S2 Fig — (DOC) [file pone.0122964.s003.doc]

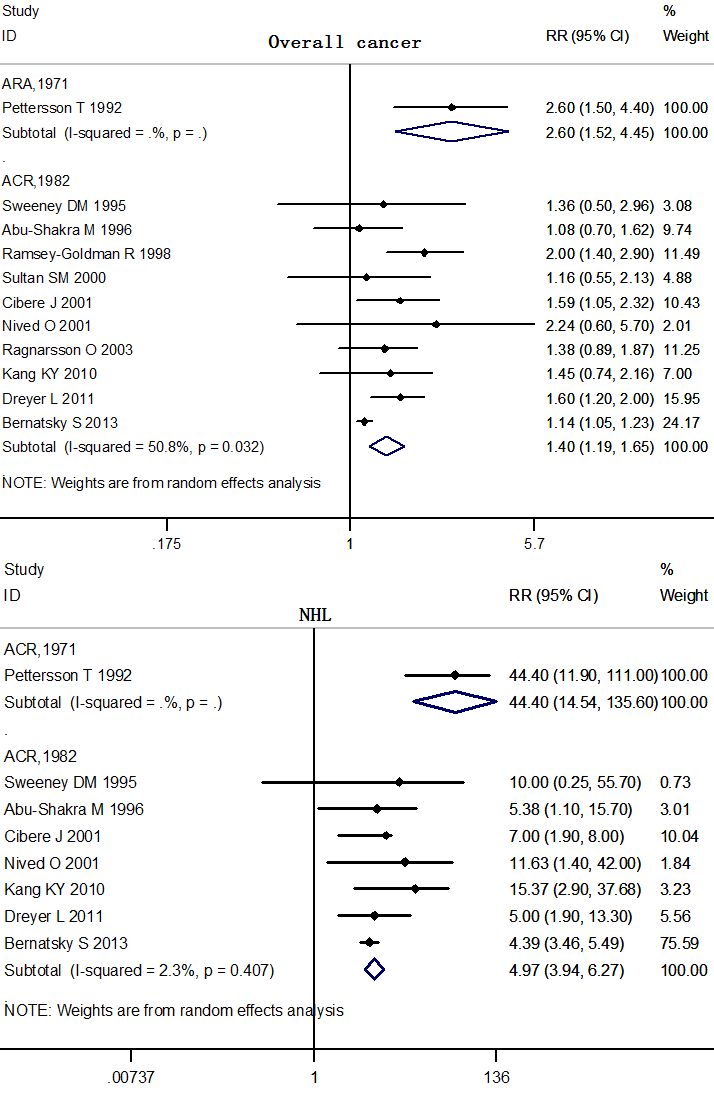

Supplement: S3 Fig — (DOC) [file pone.0122964.s004.doc]

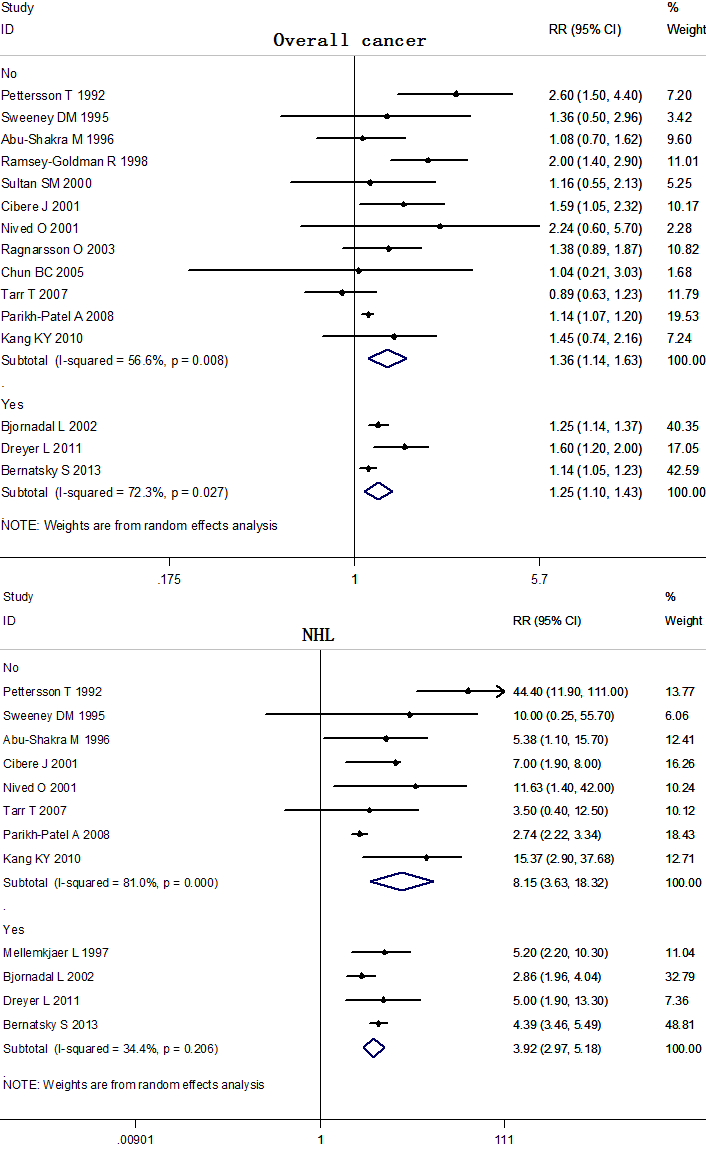

Supplement: S4 Fig — (DOC) [file pone.0122964.s005.doc]

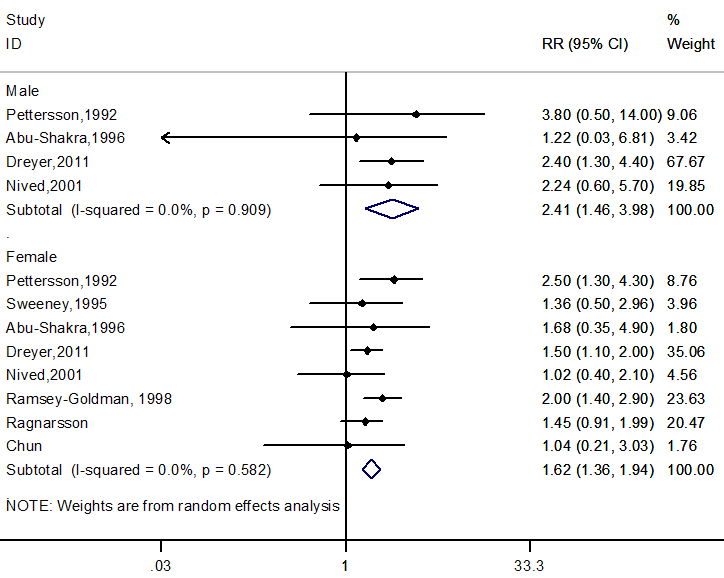

Supplement: S5 Fig — (DOC) [file pone.0122964.s006.doc]

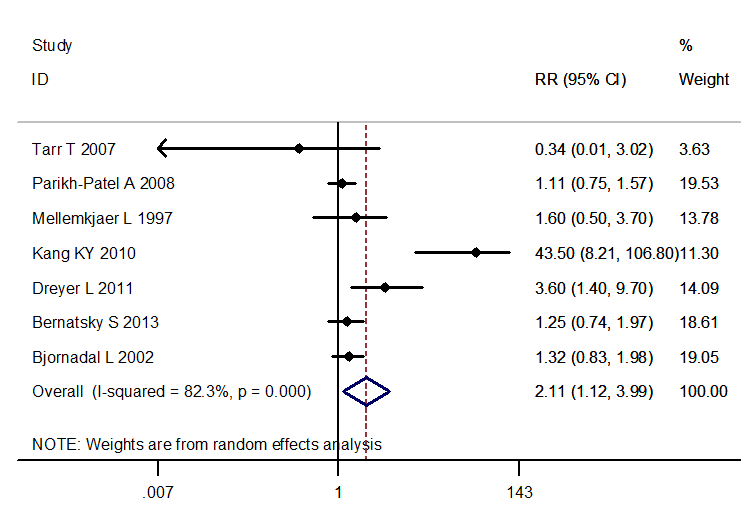

Supplement: S14 Fig — (DOC) [file pone.0122964.s015.doc]
