# Supplementary material for: Systemic Lupus Erythematous and Malignancy Risk: A Meta-Analysis
Source: PLoS One. 2015 Apr 17;10(4):e0122964. doi: 10.1371/journal.pone.0122964 (PMC4401738; doi:10.1371/journal.pone.0122964)
Supplement: S1 Table — (DOCX) [file pone.0122964.s019.docx]

|  | Selection | | | | Comparability | Outcome | | | Total score |
| --- | --- | --- | --- | --- | --- | --- | --- | --- | --- |
| Pettersson[9]  (1992, Finland) | 1 | 1 | 1 | 0 | 1 | 1 | 1 | 1 | 7 |
| Sweeney[10]  (1995, USA | 1 | 1 | 1 | 0 | 1 | 1 | 0 | 1 | 6 |
| Abu-Shakra[11]  (1996, Canada) | 1 | 1 | 1 | 1 | 1 | 1 | 1 | 1 | 8 |
| Mellemkjaer  [12] (1997  , Denmark) | 1 | 1 | 1 | 1 | 1 | 1 | 1 | 1 | 8 |
| Ramsey-Goldman[13]  (1998, USA) | 1 | 1 | 1 | 1 | 1 | 1 | 0 | 1 | 7 |
| Sultan[14]  (2000, UK) | 1 | 1 | 1 | 0 | 1 | 1 | 0 | 1 | 6 |
| Cibere[15]  (2001, Canada) | 1 | 1 | 1 | 1 | 1 | 1 | 1 | 1 | 8 |
| Nived[16]  (2001, Swenden) | 1 | 1 | 1 | 0 | 1 | 1 | 1 | 1 | 7 |
| Bjornadal[17]  (2002, Swenden) | 1 | 1 | 1 | 1 | 1 | 1 | 1 | 1 | 8 |
| Ragnarsson[18]  (2003, Iceland) | 1 | 1 | 1 | 0 | 1 | 1 | 1 | 1 | 7 |
| Chun[19]  (2005, Korea) | 1 | 1 | 1 | 0 | 1 | 1 | 0 | 1 | 6 |
| Tarr[20]  (2007, Hungary) | 1 | 1 | 1 | 0 | 1 | 1 | 1 | 1 | 7 |
| Parikh-Patel[21]  (2008, USA) | 1 | 1 | 1 | 0 | 1 | 1 | 1 | 1 | 7 |
| Kang[22]  (2010, Korea) | 1 | 1 | 1 | 0 | 1 | 1 | 1 | 1 | 7 |
| Dreyer[23]  (2011, Denmark) | 1 | 1 | 1 | 1 | 1 | 1 | 1 | 1 | 8 |
| Bernatsky[24]  (2013, Multisite) | 1 | 1 | 1 | 1 | 1 | 1 | 1 | 1 | 8 |
