# Supplementary material for: Systemic Lupus Erythematous and Malignancy Risk: A Meta-Analysis
Source: PLoS One. 2015 Apr 17;10(4):e0122964. doi: 10.1371/journal.pone.0122964 (PMC4401738; doi:10.1371/journal.pone.0122964)
Supplement: Supplementary file 4 [file pone.0122964.s007.doc]

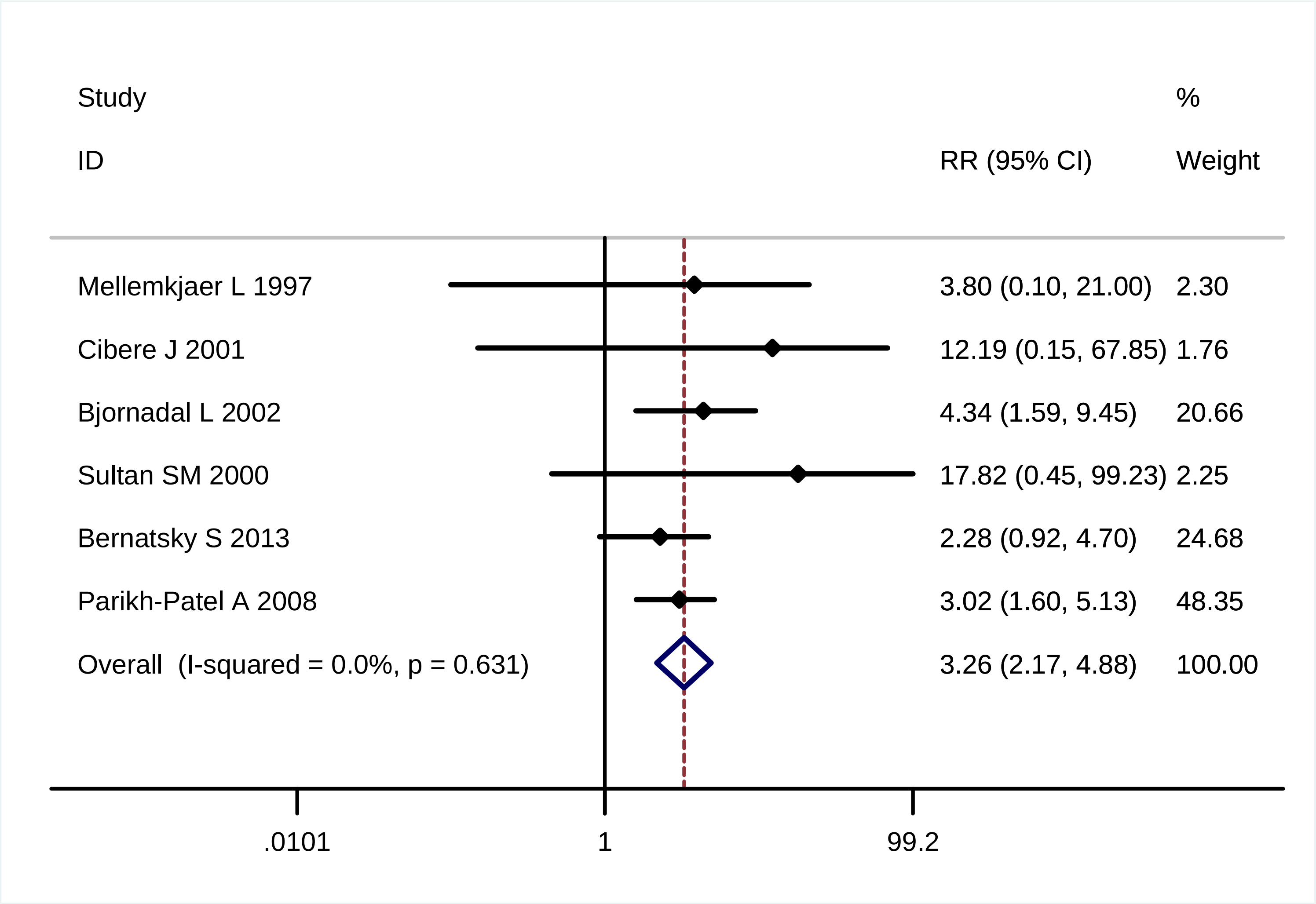
Study
ID
RR (95% CI)
%
Weight
Mellemkjaer L 1997
3.80 (0.10, 21.00)
2.30
Cibere J 2001
12.19 (0.15, 67.85)
1.76
Bjornadal L 2002
4.34 (1.59, 9.45)
20.66
Sultan SM 2000
17.82 (0.45, 99.23)
2.25
Bernatsky S 2013
2.28 (0.92, 4.70)
24.68
Parikh-Patel A 2008
3.02 (1.60, 5.13)
48.35
Overall (I-squared = 0.0%, p = 0.631)
3.26 (2.17, 4.88)
100.00
.0101
1
99.2
