# Supplementary material for: Systemic Lupus Erythematous and Malignancy Risk: A Meta-Analysis
Source: PLoS One. 2015 Apr 17;10(4):e0122964. doi: 10.1371/journal.pone.0122964 (PMC4401738; doi:10.1371/journal.pone.0122964)
Supplement: Supplementary file 5 [file pone.0122964.s008.doc]

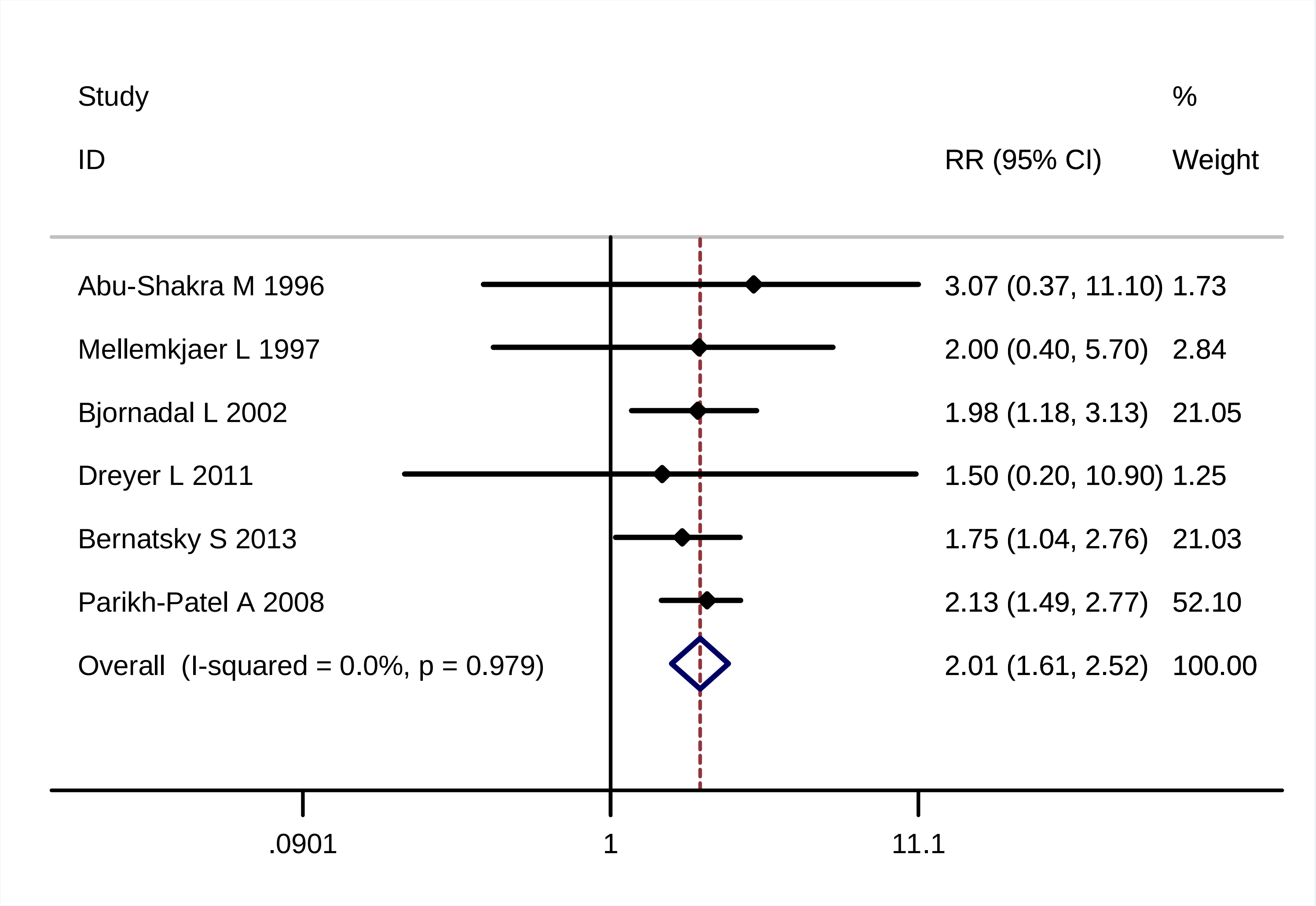
Study
ID
RR (95% CI)
%
Weight
Abu-Shakra M 1996
3.07 (0.37, 11.10) 1.73
Mellemkjaer L 1997
2.00 (0.40, 5.70) 2.84
Bjornadal L 2002
1.98 (1.18, 3.13) 21.05
Dreyer L 2011
1.50 (0.20, 10.90) 1.25
Bernatsky S 2013
1.75 (1.04, 2.76) 21.03
Parikh-Patel A 2008
2.13 (1.49, 2.77) 52.10
Overall (I-squared = 0.0%, p = 0.979)
2.01 (1.61, 2.52) 100.00
.0901
1
11.1
