# Supplementary material for: Systemic Lupus Erythematous and Malignancy Risk: A Meta-Analysis
Source: PLoS One. 2015 Apr 17;10(4):e0122964. doi: 10.1371/journal.pone.0122964 (PMC4401738; doi:10.1371/journal.pone.0122964)
Supplement: Supplementary file 6 [file pone.0122964.s009.doc]

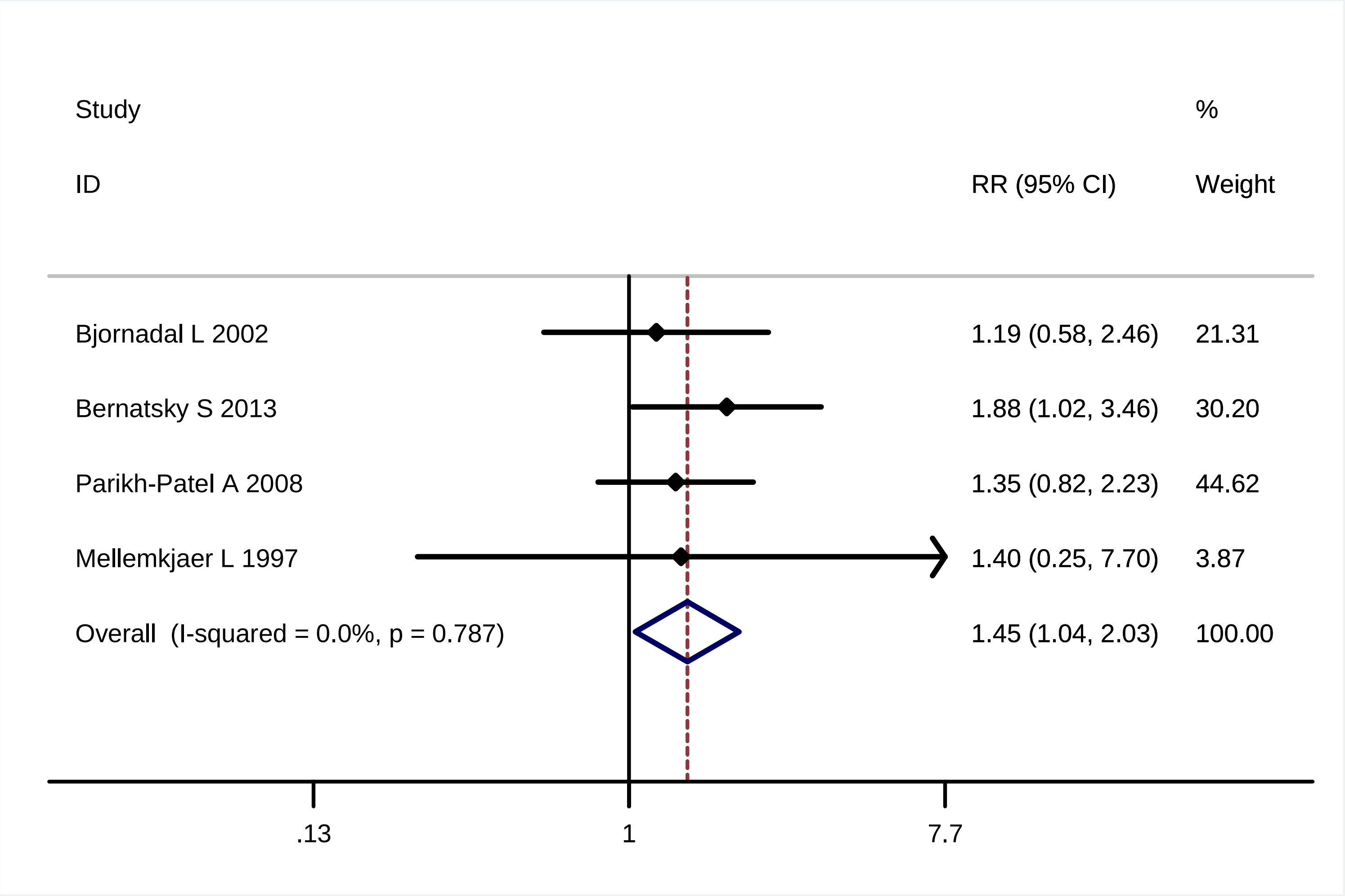
Study
ID
RR (95% CI)
%
Weight
Bjornadal L 2002
1.19 (0.58, 2.46)
21.31
Bernatsky S 2013
1.88 (1.02, 3.46)
30.20
Parikh-Patel A 2008
1.35 (0.82, 2.23)
44.62
Mellemkjaer L 1997
1.40 (0.25, 7.70)
3.87
Overall (I-squared = 0.0%, p = 0.787)
1.45 (1.04, 2.03)
100.00
.13
1
7.7
