# Supplementary material for: Systemic Lupus Erythematous and Malignancy Risk: A Meta-Analysis
Source: PLoS One. 2015 Apr 17;10(4):e0122964. doi: 10.1371/journal.pone.0122964 (PMC4401738; doi:10.1371/journal.pone.0122964)
Supplement: Supplementary file 7 [file pone.0122964.s010.doc]

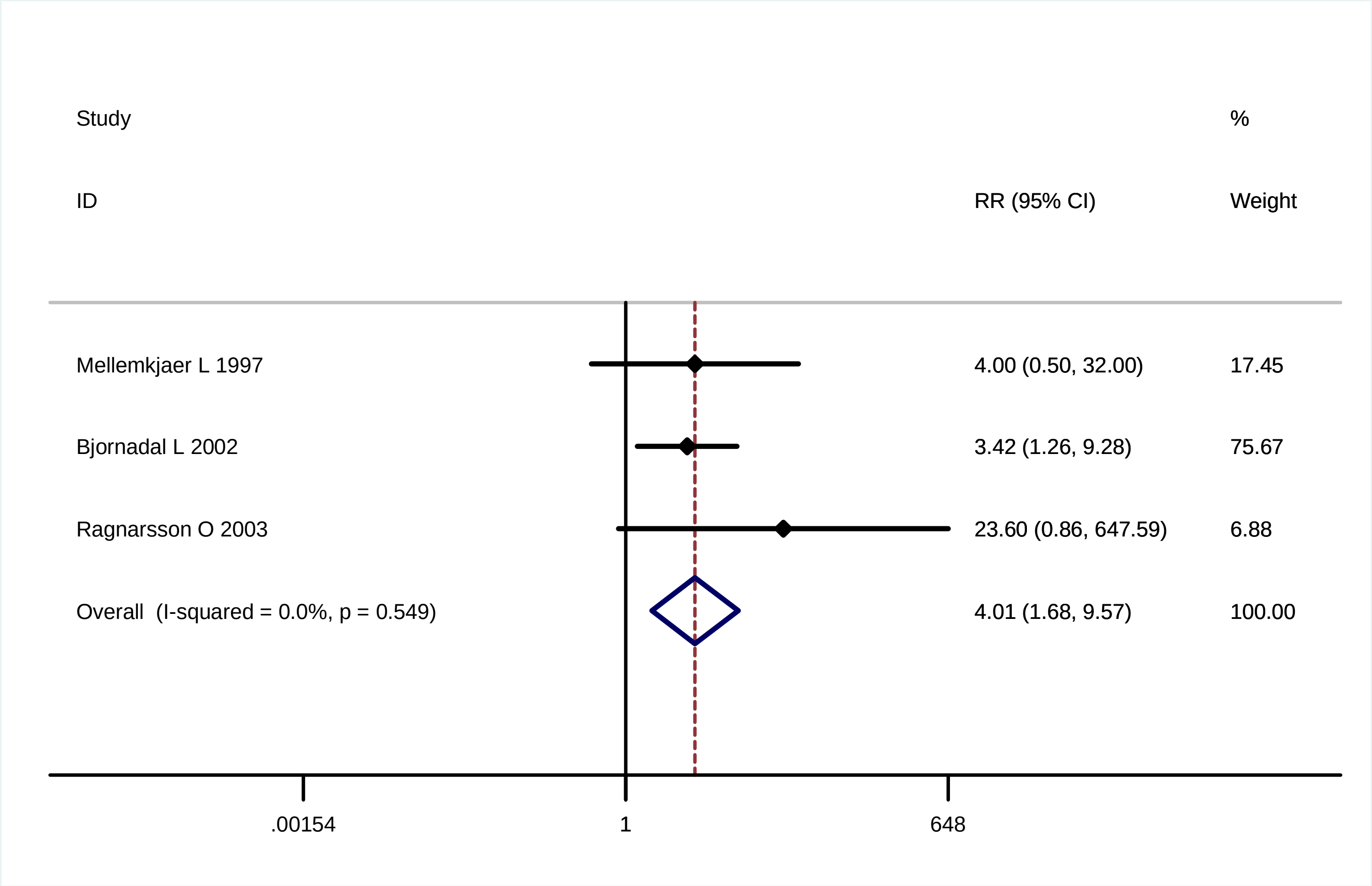
Study
ID
RR (95% CI)
%
Weight
Mellemkjaer L 1997
4.00 (0.50, 32.00)
17.45
Bjornadal L 2002
3.42 (1.26, 9.28)
75.67
Ragnarsson O 2003
23.60 (0.86, 647.59)
6.88
Overall (I-squared = 0.0%, p = 0.549)
4.01 (1.68, 9.57)
100.00
.00154
1
648
