# Supplementary material for: Systemic Lupus Erythematous and Malignancy Risk: A Meta-Analysis
Source: PLoS One. 2015 Apr 17;10(4):e0122964. doi: 10.1371/journal.pone.0122964 (PMC4401738; doi:10.1371/journal.pone.0122964)
Supplement: Supplementary file 8 [file pone.0122964.s011.doc]

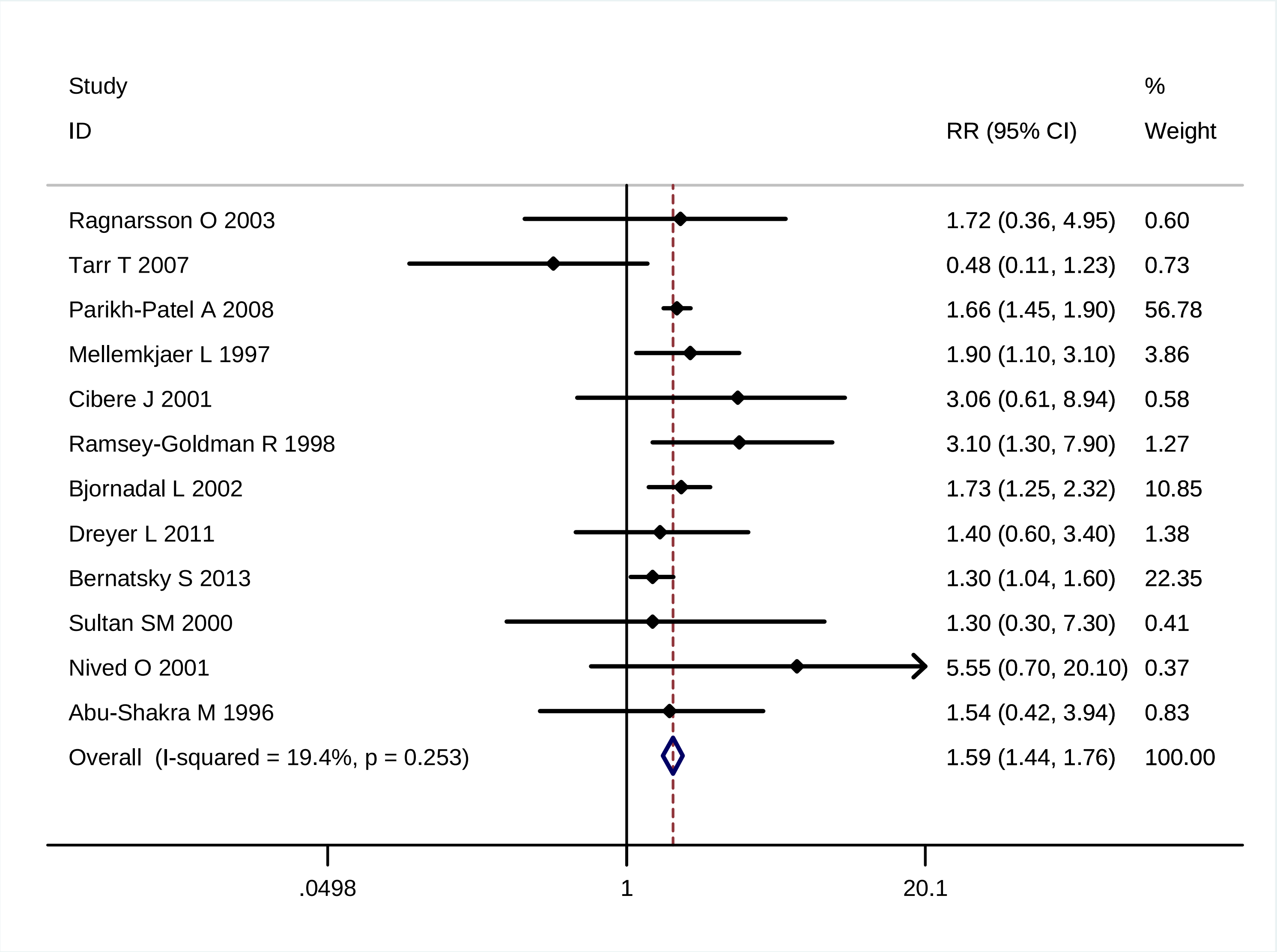
Study
ID
RR (95% CI)
%
Weight
Ragnarsson O 2003 1.72 (0.36, 4.95) 0.60
Tarr T 2007 0.48 (0.11, 1.23) 0.73
Parikh-Patel A 2008 1.66 (1.45, 1.90) 56.78
Mellemkjaer L 1997 1.90 (1.10, 3.10) 3.86
Cibere J 2001 3.06 (0.61, 8.94) 0.58
Ramsey-Goldman R 1998 3.10 (1.30, 7.90) 1.27
Bjornadal L 2002 1.73 (1.25, 2.32) 10.85
Dreyer L 2011 1.40 (0.60, 3.40) 1.38
Bernatsky S 2013 1.30 (1.04, 1.60) 22.35
Sultan SM 2000 1.30 (0.30, 7.30) 0.41
Nived O 2001 5.55 (0.70, 20.10) 0.37
Abu-Shakra M 1996 1.54 (0.42, 3.94) 0.83
Overall (I-squared = 19.4%, p = 0.253) 1.59 (1.44, 1.76) 100.00
.0498
1
20.1
