# Supplementary material for: Systemic Lupus Erythematous and Malignancy Risk: A Meta-Analysis
Source: PLoS One. 2015 Apr 17;10(4):e0122964. doi: 10.1371/journal.pone.0122964 (PMC4401738; doi:10.1371/journal.pone.0122964)
Supplement: Supplementary file 9 [file pone.0122964.s012.doc]

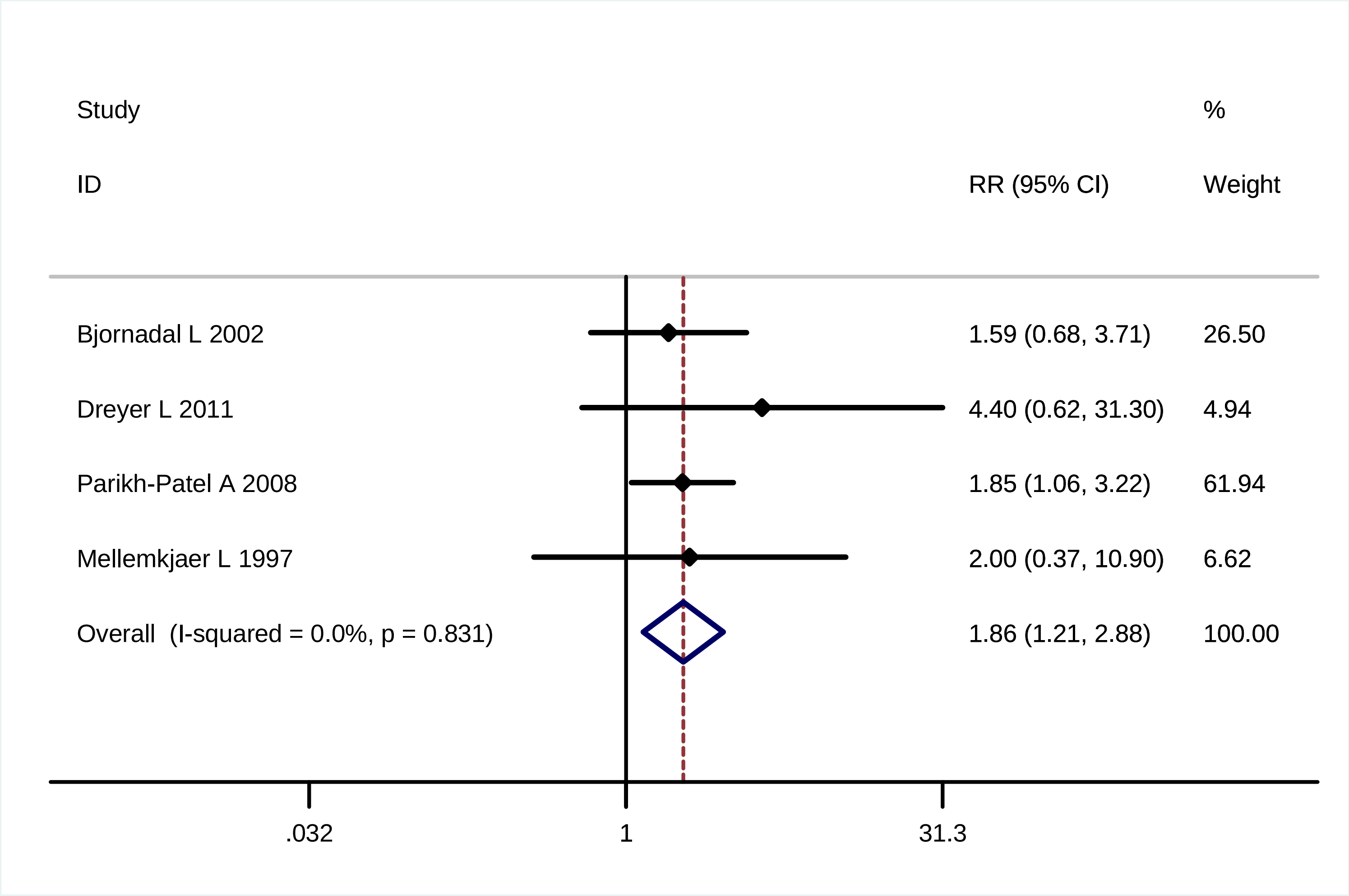

Study
ID
RR (95% CI)
%
Weight
Bjornadal L 2002
1.59 (0.68, 3.71)
26.50
Dreyer L 2011
4.40 (0.62, 31.30)
4.94
Parikh-Patel A 2008
1.85 (1.06, 3.22)
61.94
Mellemkjaer L 1997
2.00 (0.37, 10.90)
6.62
Overall (I-squared = 0.0%, p = 0.831)
1.86 (1.21, 2.88)
100.00
.032
1
31.3
