# Supplementary material for: Systemic Lupus Erythematous and Malignancy Risk: A Meta-Analysis
Source: PLoS One. 2015 Apr 17;10(4):e0122964. doi: 10.1371/journal.pone.0122964 (PMC4401738; doi:10.1371/journal.pone.0122964)
Supplement: Supplementary file 10 [file pone.0122964.s013.doc]

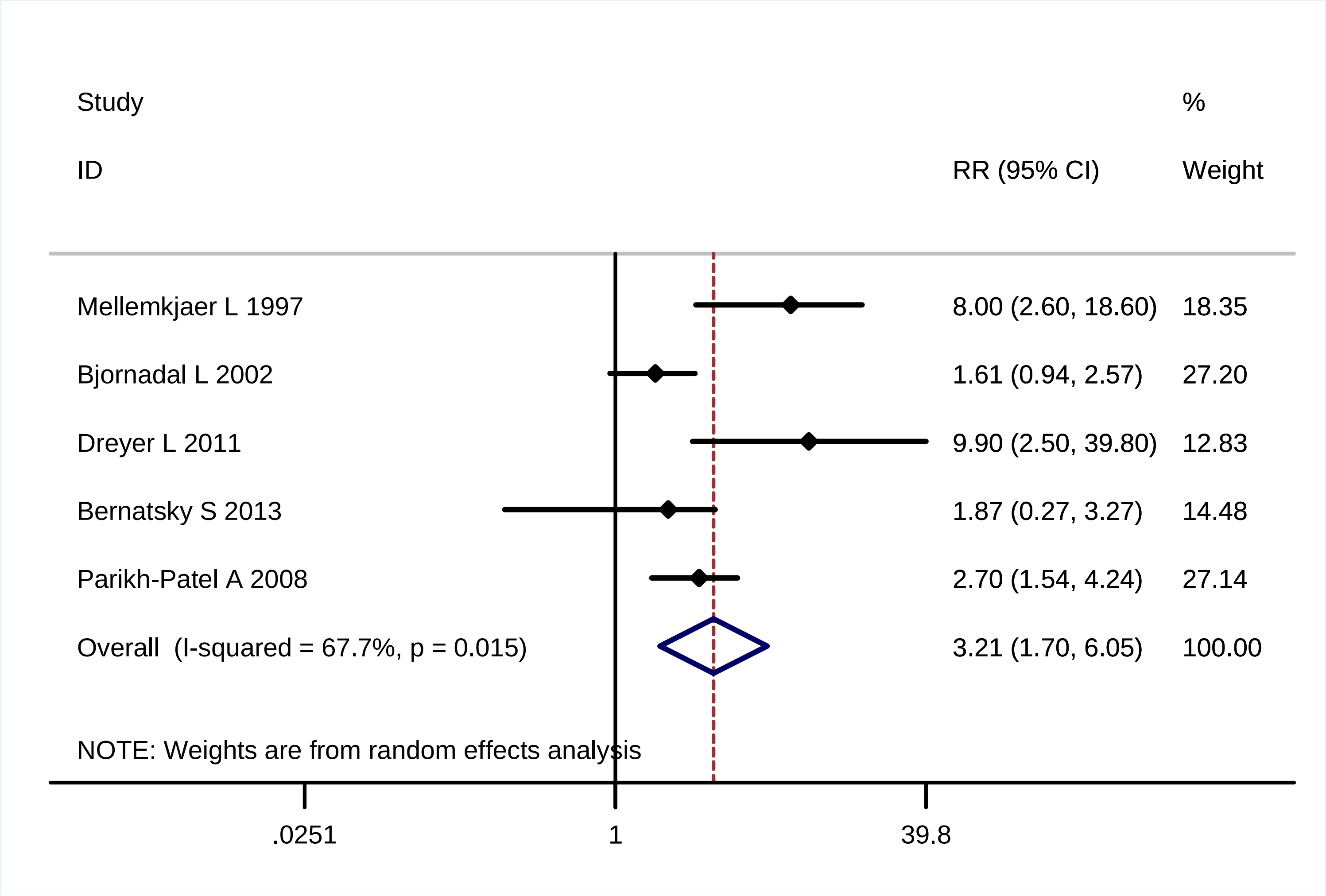
Study
ID
RR (95% CI)
%
Weight
Mellemkjaer L 1997
8.00 (2.60, 18.60)
18.35
Bjornadal L 2002
1.61 (0.94, 2.57)
27.20
Dreyer L 2011
9.90 (2.50, 39.80)
12.83
Bernatsky S 2013
1.87 (0.27, 3.27)
14.48
Parikh-Patel A 2008
2.70 (1.54, 4.24)
27.14
Overall (I-squared = 67.7%, p = 0.015)
3.21 (1.70, 6.05)
100.00
NOTE: Weights are from random effects analysis
.0251
1
39.8
