# Supplementary material for: Systemic Lupus Erythematous and Malignancy Risk: A Meta-Analysis
Source: PLoS One. 2015 Apr 17;10(4):e0122964. doi: 10.1371/journal.pone.0122964 (PMC4401738; doi:10.1371/journal.pone.0122964)
Supplement: Supplementary file 11 [file pone.0122964.s014.doc]

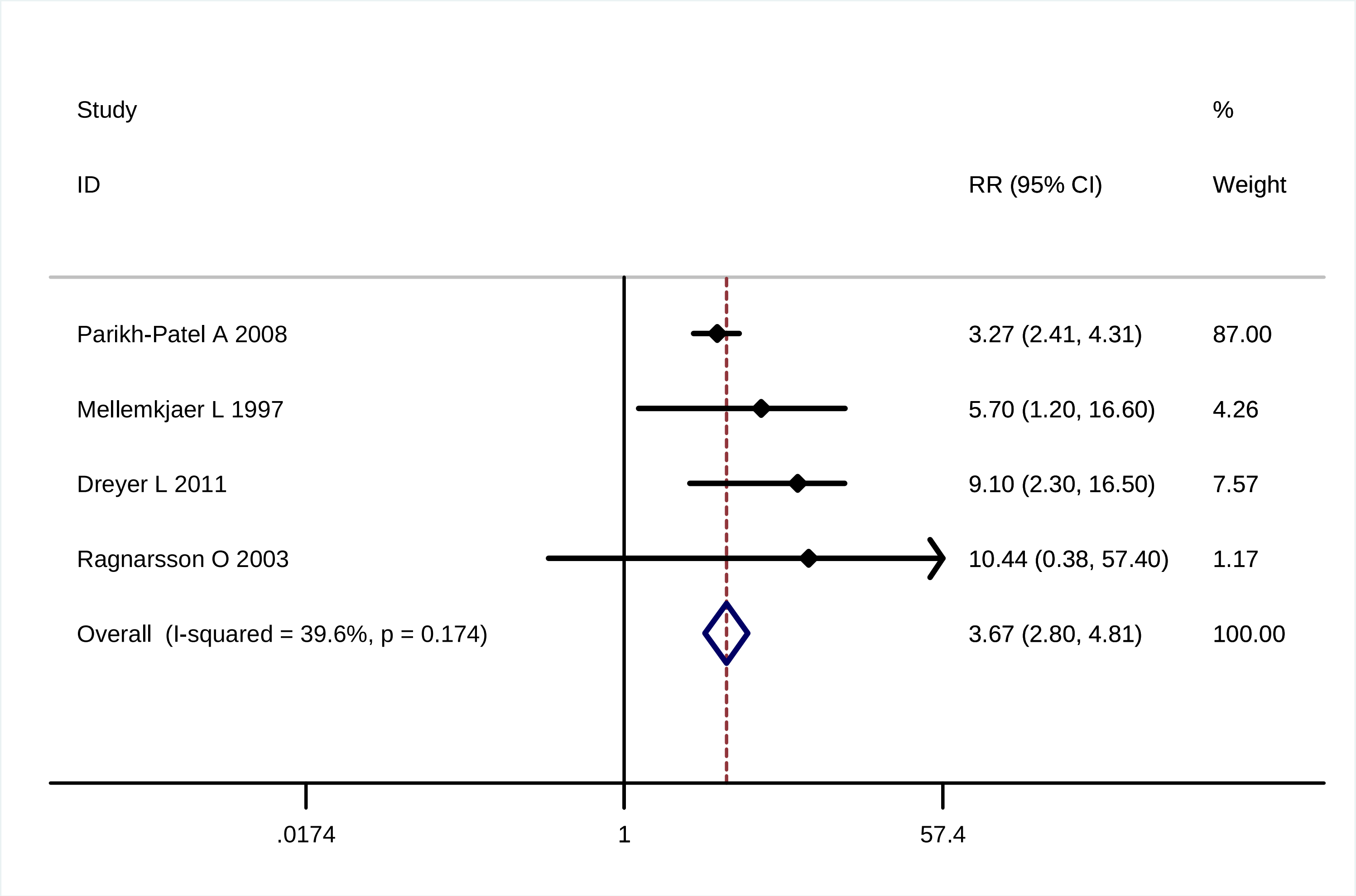
Study
%
ID
RR (95% CI)
Weight
Parikh-Patel A 2008
3.27 (2.41, 4.31)
87.00
Mellemkjaer L 1997
5.70 (1.20, 16.60)
4.26
Dreyer L 2011
9.10 (2.30, 16.50)
7.57
Ragnarsson O 2003
10.44 (0.38, 57.40)
1.17
Overall (I-squared = 39.6%, p = 0.174)
3.67 (2.80, 4.81)
100.00
.0174
1
57.4
