# Supplementary material for: Systemic Lupus Erythematous and Malignancy Risk: A Meta-Analysis
Source: PLoS One. 2015 Apr 17;10(4):e0122964. doi: 10.1371/journal.pone.0122964 (PMC4401738; doi:10.1371/journal.pone.0122964)
Supplement: Supplementary file 12 [file pone.0122964.s016.doc]

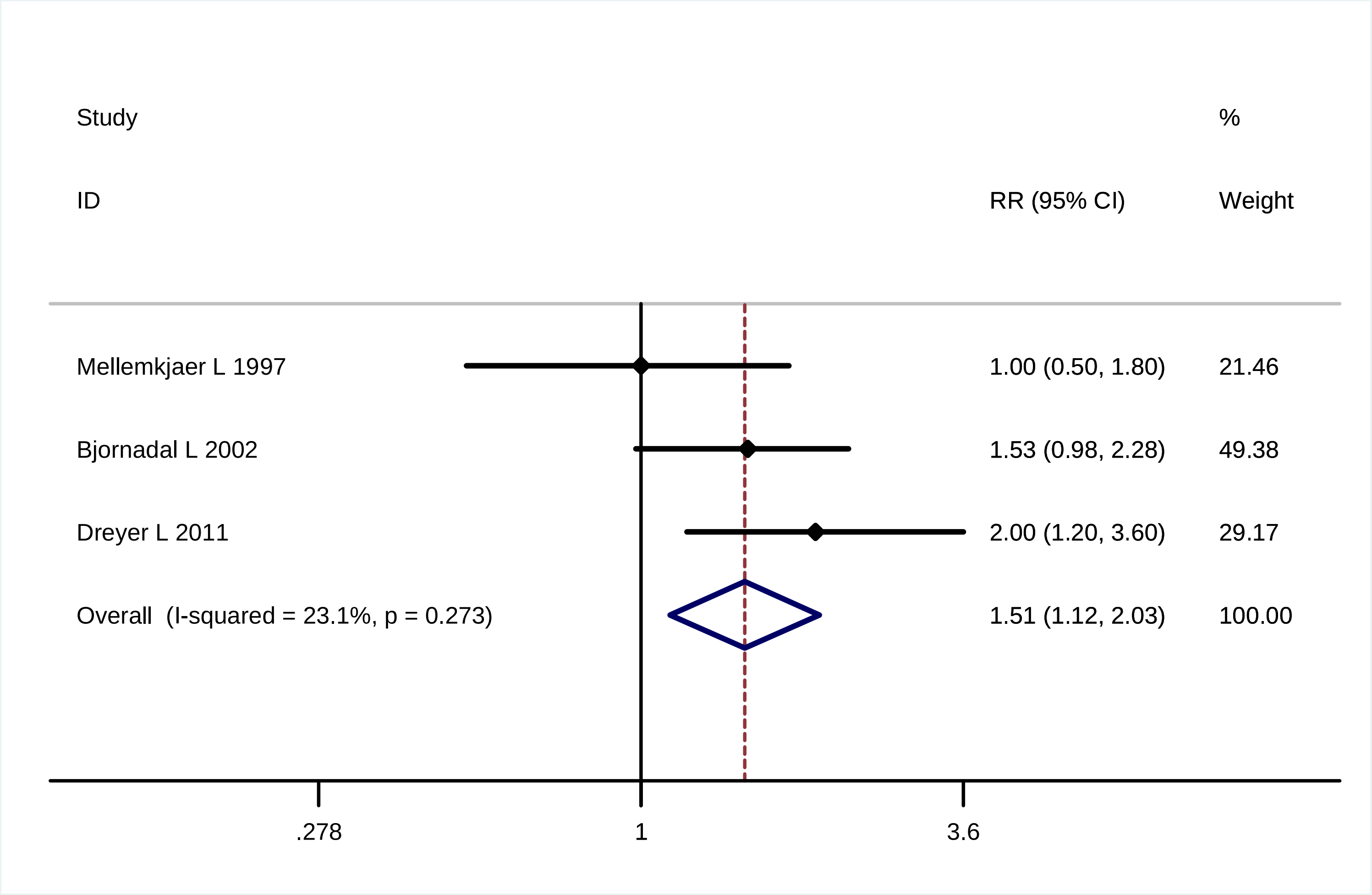
Study
%
ID
RR (95% CI)
Weight
Mellemkjaer L 1997
1.00 (0.50, 1.80)
21.46
Bjornadal L 2002
1.53 (0.98, 2.28)
49.38
Dreyer L 2011
2.00 (1.20, 3.60)
29.17
Overall (I-squared = 23.1%, p = 0.273)
1.51 (1.12, 2.03)
100.00
.278
1
3.6
