# Supplementary material for: Systemic Lupus Erythematous and Malignancy Risk: A Meta-Analysis
Source: PLoS One. 2015 Apr 17;10(4):e0122964. doi: 10.1371/journal.pone.0122964 (PMC4401738; doi:10.1371/journal.pone.0122964)
Supplement: Supplementary file 13 [file pone.0122964.s017.doc]

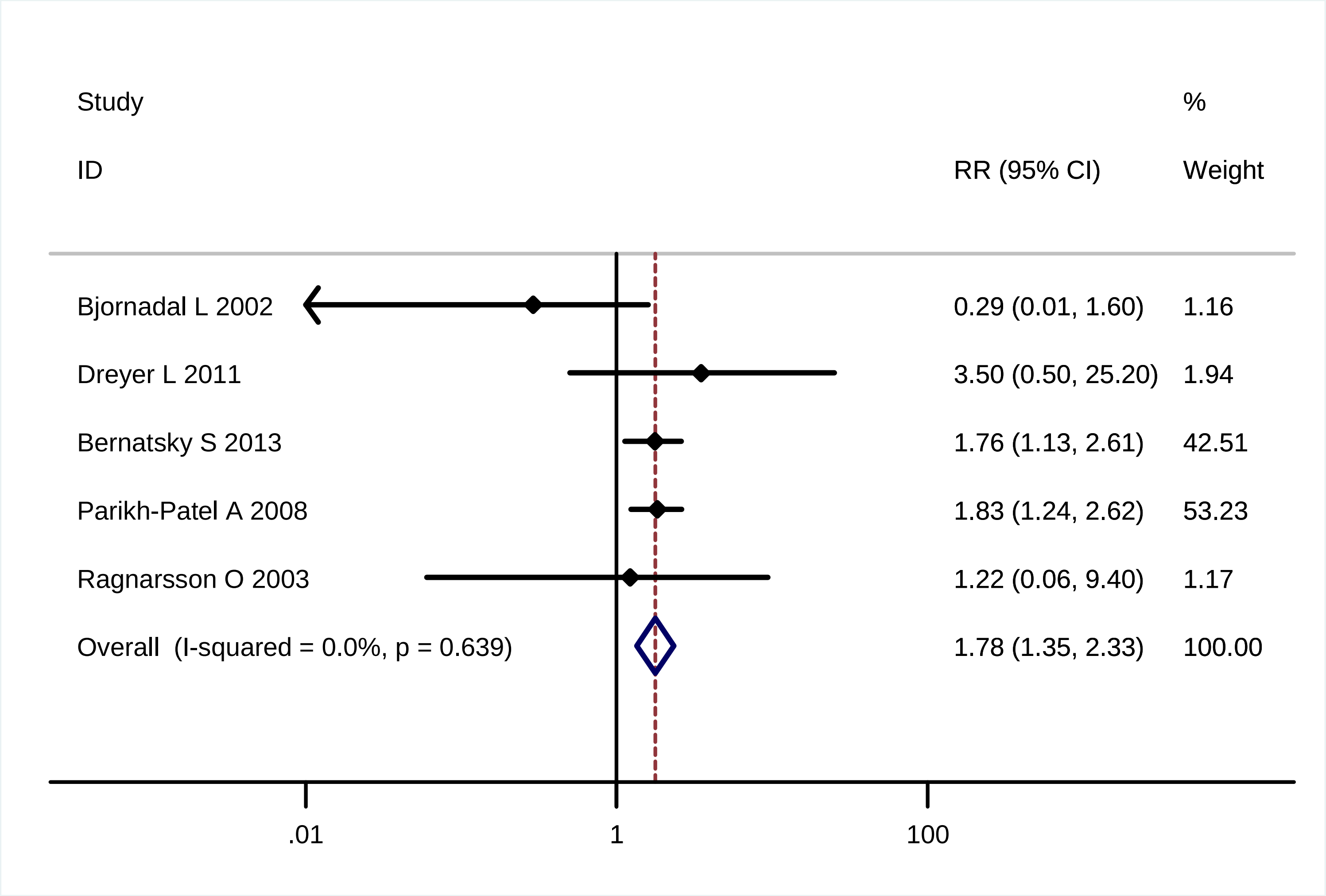

Study
ID
RR (95% CI)
%
Weight
Bjornadal L 2002
0.29 (0.01, 1.60)
1.16
Dreyer L 2011
3.50 (0.50, 25.20)
1.94
Bernatsky S 2013
1.76 (1.13, 2.61)
42.51
Parikh-Patel A 2008
1.83 (1.24, 2.62)
53.23
Ragnarsson O 2003
1.22 (0.06, 9.40)
1.17
Overall (I-squared = 0.0%, p = 0.639)
1.78 (1.35, 2.33)
100.00
.01
1
100
