# Supplementary material for: Systemic Lupus Erythematous and Malignancy Risk: A Meta-Analysis
Source: PLoS One. 2015 Apr 17;10(4):e0122964. doi: 10.1371/journal.pone.0122964 (PMC4401738; doi:10.1371/journal.pone.0122964)
Supplement: Supplementary file 14 [file pone.0122964.s018.doc]

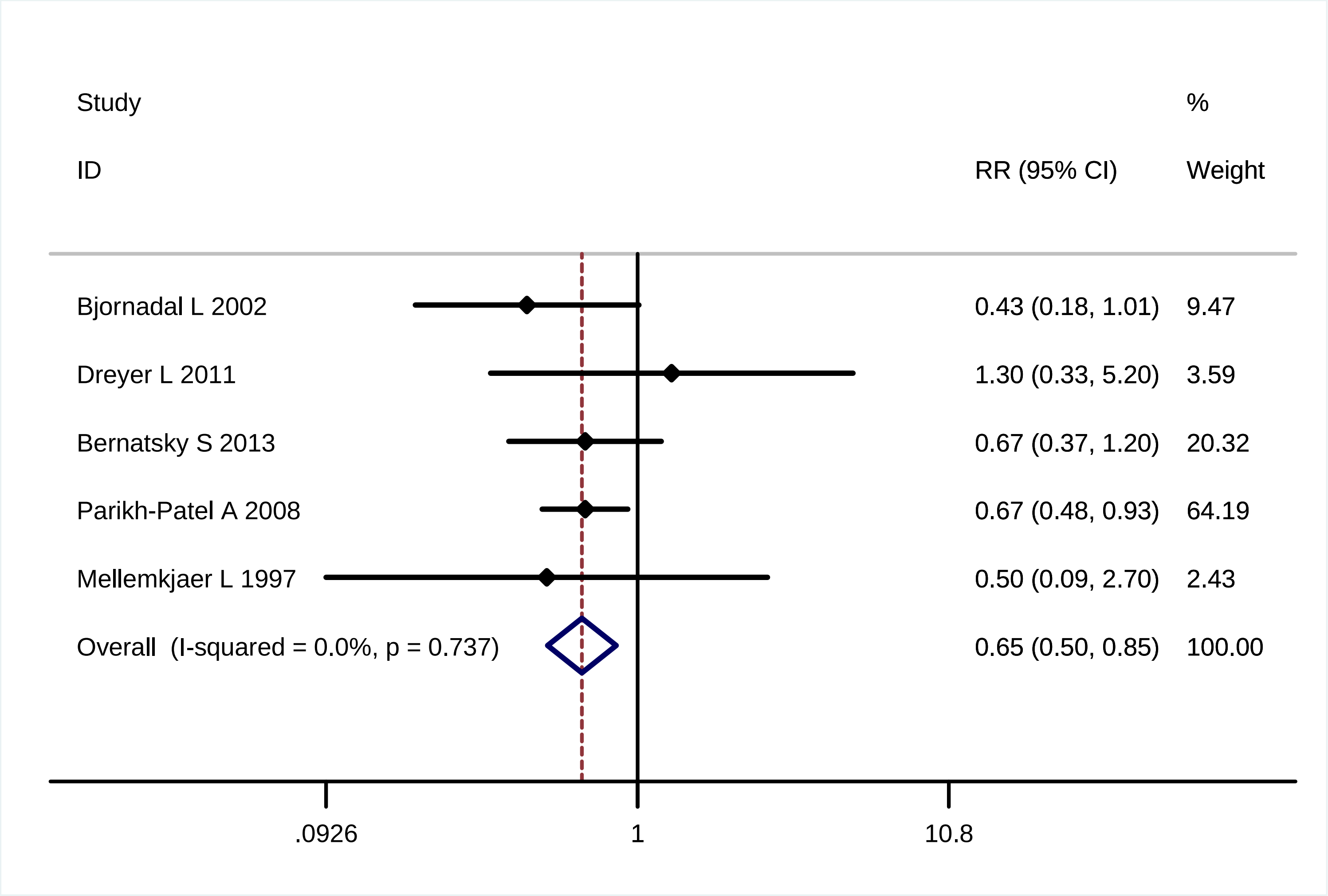
Study
ID
RR (95% CI)
%
Weight
Bjornadal L 2002
0.43 (0.18, 1.01)
9.47
Dreyer L 2011
1.30 (0.33, 5.20)
3.59
Bernatsky S 2013
0.67 (0.37, 1.20)
20.32
Parikh-Patel A 2008
0.67 (0.48, 0.93)
64.19
Mellemkjaer L 1997
0.50 (0.09, 2.70)
2.43
Overall (I-squared = 0.0%, p = 0.737)
0.65 (0.50, 0.85)
100.00
.0926
1
10.8
